# Emergency medical services personnel’s work capability sets: associations with job demands, job resources, and flourishing at work

**DOI:** 10.3389/fpsyg.2026.1909957

**Published:** 2026-10-26

**Authors:** Hazel Michellé Hattingh, Neil Bertrand Barnard, Lynelle Coxen

**Affiliations:** 1 Optentia Research Unit, North-West University, Vanderbijlpark, South Africa; 2 School of Industrial Psychology and Human Resource Management, Faculty of Economic and Management Sciences, North-West University, Vanderbijlpark, South Africa

**Keywords:** emergency medical services personnel, flourishing at work, job demands, job resources, latent class analysis, negative affect, sustainable employability model, work capabilities

## Abstract

Emergency Medical Services (EMS) personnel perform complex and demanding work under challenging conditions while providing essential healthcare services. Understanding the factors that support their flourishing at work is important for their sustainable employability and the functioning of healthcare systems. This study investigated EMS personnel’s work capability sets (enabled and achieved valued work outcomes) and their associations with job demands (emotional demands and work pressure), job resources (autonomy at work and co-worker support), flourishing at work (emotional, psychological, and social well-being), and negative affect. A cross-sectional survey design was used with 197 EMS personnel in South Africa. Participants completed the Job Demands-Resources Questionnaire, the Capability Set for Work Questionnaire, and the Flourishing at Work-Short Form. Correlation analysis, confirmatory factor analysis, latent class analysis, and Bolck-Croon-Hagenaars analyses were conducted. Using knowledge and skills, developing knowledge and skills, and building and maintaining meaningful relationships at work emerged as the strongest work capabilities, whereas earning a good income, involvement in important decisions, and contributing to something valuable were the weakest. Latent class analysis identified three distinct capability sets: a weak capability set (48%), a constrained capability set (30%), and a robust capability set (22%). Emotional demands were negatively associated with work capabilities and flourishing and positively associated with negative affect, whereas work pressure was positively associated with negative affect but not significantly associated with overall work capabilities or flourishing. Job demands did not significantly differentiate the capability sets, whereas autonomy and co-worker support did. EMS personnel in the robust capability set reported greater flourishing and lower negative affect than those in the weak capability set. The findings are consistent with the Sustainable Employability model grounded in the Capability Approach showing that broader capability sets are associated with greater flourishing at work. Strengthening enabling work conditions, particularly autonomy and co-worker support, while attending to demands that may constrain work capabilities, may help create conditions conducive to the realization of valued work outcomes, flourishing, and sustainable employability among EMS personnel.

## 1 Introduction

### 1.1 Background

Emergency Medical Services (EMS) personnel perform complex and demanding work under challenging circumstances. They are frequently exposed to traumatic incidents, potential violence, and high job demands while providing essential healthcare services that are critical to the functioning of healthcare systems and the well-being of society (Farcas et al., 2024; Mishra et al., 2010; Thielmann et al., 2022; Van Puyvelde et al., 2023). In South Africa, these challenges are compounded by broader socio-economic conditions, including economic constraints, high crime rates, and unemployment (Krugel, 2025). Consequently, EMS personnel are required to deliver life-saving care within environments that may place considerable strain on their well-being and long-term functioning. Given the vital role they play in healthcare delivery, understanding how to support their sustained flourishing at work is an important area of inquiry.

The sustainable employability (SE) model (Van der Klink et al., 2016), grounded in the capability approach (CA) (Sen, 1985), offers a useful framework for examining how employees can maintain their well-being and functioning over time. Central to this perspective is the notion that employees require genuine opportunities to achieve work outcomes they have reason to value. The job demands-resources (JD-R) model (Demerouti et al., 2001) complements this perspective by explaining how characteristics of the work environment may either support or hinder employees’ ability to function effectively. Drawing on these frameworks, the present study examined EMS personnel’s flourishing at work by investigating the associations between their job demands, job resources, and work capabilities, with the aim of advancing understanding of their SE.

### 1.2 Work context of EMS personnel

Emergency Medical Services (EMS) represent an integrated system that coordinates personnel, infrastructure, and equipment to provide timely and effective medical care to individuals experiencing sudden illness or injury (Al-Shaqsi, 2010). Within this system, EMS personnel (including basic ambulance assistants, emergency care assistants, emergency care technicians, paramedics, and emergency care practitioners) deliver life-saving interventions at emergency scenes and continue treatment during patient transport to healthcare facilities (Al-Shaqsi, 2010; Health Professions Council of South Africa, 2026).

EMS personnel fulfil critical functions at individual, healthcare system, and societal levels. Their rapid assessment and intervention directly influence patient survival and recovery outcomes (Cunningham et al., 2022), while their ability to stabilize patients and facilitate access to hospital care contributes to continuity of care within healthcare systems (Newton et al., 2024). In South Africa, access to emergency medical treatment is constitutionally protected under Section 27 of the Constitution (1996), positioning EMS personnel at the intersection of healthcare delivery, social justice, and health equity (Farcas et al., 2024). By promoting access to urgent healthcare, EMS systems also contribute to broader health objectives, including the United Nations Sustainable Development Goal 3 (Good Health and Well-Being) and the African Union Agenda 2063 Goal 3 (Healthy and Well-Nourished Citizens), both of which emphasize universal access to quality health services and strengthened health systems (African Union, 2026; United Nations, 2015).

Despite their essential societal role, EMS personnel work in environments characterized by uncertainty, time pressure, and considerable emotional and physical demands. Their work requires rapid decision-making in response to medical crises, severe injuries, and large-scale emergencies (Elmqvist et al., 2010; Karrasch et al., 2020). Exposure to severe injury, death, violence, and infection risks is a routine aspect of their work, making trauma exposure an inherent feature of the profession rather than an exceptional occurrence (Elsässer et al., 2024; Thielmann et al., 2022).

EMS personnel constitute a distinct occupational group with unique patterns of trauma exposure and occupational stress (Arjmand et al., 2024). Unlike many other trauma-exposed professions, they typically encounter traumatic events in civilian settings, including motor vehicle accidents, violent assaults, suicides, and pediatric emergencies (Arjmand et al., 2024). Furthermore, many EMS personnel live and work within the same communities in which emergencies occur, potentially intensifying emotional demands by blurring the boundaries between their professional and personal lives (Jones et al., 2018).

The demands of EMS work extend beyond exposure to traumatic events. EMS personnel also contend with chronic organizational and operational stressors, including physically demanding tasks, labor-management tensions, inadequate equipment, staffing shortages, extended shifts, mandated overtime, and high workloads (Haugen et al., 2012; Jones et al., 2018). The combination of acute trauma exposure and persistent organizational pressures creates a layered burden that distinguishes EMS work from many other occupations.

In South Africa, these occupational challenges are further amplified by broader socio-economic and structural conditions. EMS personnel frequently operate in environments characterized by high crime rates, economic instability, and resource constraints (Krugel, 2025). Research suggests that ambulance personnel in South Africa may experience post-traumatic stress symptoms at rates as high as 30%, substantially exceeding the prevalence typically reported among emergency workers internationally (Alzahrani et al., 2025; Ntatamala and Adams, 2022; Petrie et al., 2018). At the same time, workforce projections indicate that approximately 96,000 additional EMS providers will be required by 2030 to meet national service demands, while shortages of personnel, vehicles, and equipment continue to increase pressure on existing staff (Tiwari et al., 2021).

The cumulative effects of these demands have been associated with burnout, post-traumatic stress symptoms, somatic complaints, and diminished capacity to provide high-quality patient care (Elsässer et al., 2024; Karrasch et al., 2020; Wojtysiak et al., 2021). Such outcomes threaten not only the well-being of EMS personnel but also the sustainability of emergency healthcare delivery. A critical paradox therefore emerges: while EMS personnel are essential to population health and healthcare system resilience, the conditions under which they work may undermine their own long-term functioning and well-being. Understanding how to support their sustained flourishing and employability is therefore an important area of investigation.

### 1.3 Promoting EMS personnel’s well-being at work

EMS organizations often face challenges in creating work environments that adequately support employee well-being. Mental health concerns are particularly prominent among EMS personnel, who are reportedly twice as likely to attribute psychological distress to work-related factors than employees in other occupations (Johnston et al., 2025). In some organizations, poor mental health and unsupportive workplace cultures have contributed to workforce losses of up to 50%, placing both workforce stability and service delivery at risk (Johnston et al., 2025). Although many EMS organizations have introduced wellness initiatives such as counselling services and mindfulness programs, employee participation has often been limited and improvements in well-being remain modest (Johnston et al., 2025).

These findings highlight the distinction between individual wellness initiatives and broader workplace well-being. While wellness programs may help employees cope with work-related stressors, they rarely address the underlying characteristics of work that contribute to strain. Workplace well-being is shaped by how work is designed, organized, and managed, including factors such as workload, work environments, interpersonal relationships, and organizational culture (De Neve and Ward, 2025). Sustainable improvements in employee well-being therefore require attention to the broader conditions under which work is performed, with the aim of creating work environments that are fair, meaningful, and manageable.

Promoting employee well-being is important not only for employees themselves but also for the effectiveness of healthcare organizations. Within healthcare systems, employee well-being has been linked to improved patient safety, higher quality of care, and stronger organizational performance (Ericsson et al., 2022; Kirk, 2024; Regehr and Millar, 2007; Stassen et al., 2013). It has also been associated with greater resilience, meaning at work, and job satisfaction, while reducing turnover intentions and supporting workforce stability (Bono et al., 2011; Clark et al., 2021; Gayton and Lovell, 2012; Mason, 2024). Consequently, there is increasing recognition that sustainable healthcare systems depend not only on patient-centered care but also on work environments that promote employee dignity, participation, and sustainable job quality (De Neve and Ward, 2025).

For EMS personnel, promoting well-being therefore requires more than isolated wellness initiatives. It requires understanding and addressing the conditions that enable employees to function effectively and sustain their well-being over time. Supporting EMS personnel’s long-term functioning is essential not only for their own well-being but also for workforce retention, service continuity, and healthcare system resilience. These considerations highlight the importance of examining the SE of EMS personnel.

### 1.4 Sustainable employability of EMS personnel

SE, broadly defined as employees’ ability and motivation to remain in work throughout their working lives, has received increasing scholarly attention in recent years (Gürbüz et al., 2023). From an organizational perspective, maintaining a sustainably employable workforce is important because it helps reduce the costs associated with turnover, absenteeism, and burnout (De Jonge and Peeters, 2019). Achieving SE requires organizations to create work environments that enable employees to maintain their health, optimal functioning, and well-being over time (Van der Klink et al., 2016; Van der Klink and Rothmann, 2026). This is particularly important in EMS organizations, where workforce sustainability directly affects healthcare system functioning and societal resilience.

Recent scholarship has increasingly adopted value-based perspectives on SE, recognizing that a sustainable working life depends not only on employees’ ability to continue working but also on their opportunities to engage in work they value (Meerman et al., 2022). The capability approach (CA) (Sen, 1985) provides a useful framework for understanding these opportunities. This perspective is grounded in three principles: normativity, which emphasizes improving working lives so that individuals can flourish sustainably; diversity, which acknowledges that employees differ in what they value in their work; and contextuality, which recognizes that opportunities to achieve valued work outcomes are shaped by the conditions of the work environment (Meerman et al., 2022).

From a capability perspective, SE depends not only on what employees achieve, but also on their genuine opportunities to achieve work outcomes they have reason to value (Van der Klink and Rothmann, 2026). SE emerges when employees regard particular work outcomes as important (importance), have opportunities within their work environments to pursue these outcomes (enablement), and are able to realize them in practice (achievement) (Gürbüz et al., 2023; Van der Klink et al., 2026; Van der Klink et al., 2016; Van der Klink and Rothmann, 2026).

Among the various conceptualizations of SE, the model proposed by Van der Klink et al. (2016) is widely regarded as one of the most comprehensive (Gürbüz et al., 2023). Grounded in the CA, this model conceptualizes SE as employees’ ability to realize valuable opportunities through a set of work capabilities while maintaining their health and well-being throughout their working lives (Van der Klink et al., 2026; Van der Klink and Rothmann, 2026). Within this framework, work capabilities emerge through the interaction of work values, resources, and conversion factors that enable employees to transform available resources into opportunities for achieving valued work outcomes (Gürbüz et al., 2023). The model further proposes that these opportunities are reflected in work-related outcomes (or functionings) such as flourishing at work, while stronger capability sets are generally associated with higher well-being and fewer negative outcomes (Gürbüz et al., 2023; Rothmann et al., 2026a; Rothmann et al., 2026b).

For EMS personnel, the concept of SE is particularly relevant given the demanding and often traumatic conditions under which they work. The opportunities available to achieve valued work outcomes may be shaped by the unique demands and resources of the EMS environment, influencing both their well-being and their long-term participation in the profession (Arjmand et al., 2024; Meerman et al., 2022). Understanding these dynamics is therefore essential for supporting EMS personnel’s sustained flourishing and employability.

### 1.5 EMS personnel’s means to achieve

The demanding nature of EMS work highlights the importance of understanding the contextual conditions that enable employees to achieve valued work outcomes. Within the SE framework grounded in the CA, employees require resources that can be converted into genuine opportunities to realize what they value in their work (Van der Klink et al., 2026). These resources include both job demands and job resources, which together shape employees’ opportunities to achieve valued work goals.

Importantly, from a capability perspective, job characteristics are not inherently beneficial or detrimental. Even demanding aspects of work may contribute to valued outcomes when they align with what employees regard as meaningful (Gürbüz et al., 2023). For EMS personnel, emotional demands and time pressure may be experienced as worthwhile when they are associated with saving lives and contributing to society. However, when demands exceed available support and resources, they may restrict employees’ opportunities to achieve valued work outcomes (Ragadu and Rothmann, 2026).

The capability perspective further distinguishes between resources and conversion factors. While resources provide employees with potential means to achieve valued outcomes, conversion factors determine whether these resources can be transformed into real opportunities (Gürbüz et al., 2023; Van der Klink et al., 2026). Consequently, the availability of resources alone does not guarantee SE. Employees must also be able and enabled to convert available resources into work capabilities (Van der Klink and Rothmann, 2026).

The Job Demands-Resources (JD-R) theory (Demerouti et al., 2001) provides a complementary explanation of how work characteristics influence employee functioning. According to this perspective, job demands may contribute to strain through health-impairment processes, whereas job resources may buffer the effects of demands and stimulate motivational processes that enhance well-being and performance (Bakker and Demerouti, 2024; Tummers and Bakker, 2021). When integrated with the SE framework, the JD-R perspective suggests that job demands and job resources influence employees’ opportunities to develop and realize work capabilities.

Empirical studies combining the JD-R and SE frameworks indicate that job demands and job resources jointly shape employees’ work capability sets (Abma et al., 2016; Barnard et al., 2023b; Mnxuma et al., 2026; Ragadu and Rothmann, 2026). In particular, employees working in high-demand, low-resource environments tend to report weaker capability sets than those working in demanding contexts where adequate resources are available (Barnard et al., 2023b). These findings suggest that the availability of resources may play an important role in sustaining employees’ opportunities to achieve valued work outcomes despite challenging work conditions.

Two job resources that may be particularly relevant in the EMS context are autonomy at work and co-worker support. Autonomy refers to employees’ discretion in carrying out their work and making work-related decisions, whereas co-worker support refers to the assistance, encouragement, and sense of being valued that employees receive from colleagues (Bakker et al., 2005). Both resources have been associated with stronger work capability sets and improved well-being (Barnard et al., 2023b). Autonomy may reduce the strain associated with demanding work conditions and enhance affective well-being (Laguna et al., 2022; Tahar et al., 2023), while co-worker support has been linked to improved mental health, job satisfaction, and workplace functioning (Marange et al., 2024).

For EMS personnel, limited autonomy and insufficient co-worker support may constrain opportunities to achieve valued work outcomes, whereas adequate resources may enable employees to navigate demanding work environments while maintaining their ability to realize what they value in their work. Within the SE framework, these opportunities are reflected in employees’ work capability sets. Accordingly, the present study examined emotional demands and work pressure as job demands, and autonomy at work and co-worker support as job resources, and investigated how these work characteristics are associated with EMS personnel’s work capabilities.

### 1.6 Work capabilities of EMS personnel

Within the SE framework, work capabilities represent employees’ real opportunities to achieve work outcomes they value (Van der Klink et al., 2026; Van der Klink and Rothmann, 2026). These opportunities emerge when employees regard particular work outcomes as important, are provided with opportunities to pursue them, and are able to realize them in practice (Abma et al., 2016). The resulting capability set reflects the range of valued work outcomes that employees are both able and enabled to achieve within their work environments.

To operationalize this framework, Abma et al. (2016) identified seven work values that are widely regarded as important in working life: (1) using knowledge and skills, (2) developing knowledge and skills, (3) involvement in important decisions, (4) building and maintaining meaningful relationships at work, (5) setting one’s own goals, (6) earning a good income, and (7) contributing to something valuable. Together, these capabilities provide an indication of employees’ opportunities to achieve forms of work and work outcomes they value (Van der Klink et al., 2026).

Research consistently demonstrates that broader capability sets are associated with more positive work outcomes. Employees with stronger capability sets report higher levels of work engagement, work ability, performance, and well-being, while experiencing lower vulnerability to burnout and other adverse outcomes (Abma et al., 2016; Gürbüz et al., 2023; Rothmann et al., 2026a; Rothmann et al., 2026b). Conversely, resource-constrained or precarious work environments may restrict employees’ opportunities to realize valued work outcomes, thereby undermining sustainable functioning at work (Barnard et al., 2023b; Ragadu and Rothmann, 2026).

A person-centered perspective provides additional insight into how work capabilities are experienced within occupational groups. Rather than assuming that employees experience similar opportunities, person-centered approaches recognize that distinct sub-populations may exist based on different configurations of work capabilities (Spurk et al., 2020). Within the healthcare sector, among a sample of emergency nurses, three capability sets were identified (i.e., robust, inadequate, and weak), with differing levels of resources and work functioning (Barnard et al., 2023a). Similar patterns have been reported among industrial psychology and human resource professionals, special education teachers, and early childhood development practitioners, where employees differed in their opportunities to achieve valued work outcomes despite working within the same occupational contexts (Mnxuma et al., 2026; Murangi et al., 2022; Ragadu and Rothmann, 2023). These findings suggest that employees within the same profession may experience substantially different levels of SE depending on the opportunities available to them.

Such differences may be particularly relevant in the EMS context. EMS personnel work in environments characterized by unpredictability, exposure to trauma, and potential threats to personal safety, including robbery, violence, and hijacking (Malamule, 2026; Wende, 2024). Furthermore, EMS work is inherently time-critical, emotionally demanding, and often performed under conditions of uncertainty (Gayton and Lovell, 2012; Murray et al., 2020; Regehr and Millar, 2007). These contextual realities may shape EMS personnel’s opportunities to achieve valued work outcomes and, consequently, the configuration of their work capability sets.

Accordingly, the present study adopted a person-centered approach to examine EMS personnel’s work capability sets. Specifically, it sought to identify latent sub-populations characterized by distinct capability configurations and to examine how these groups differed in terms of their job demands, job resources, and flourishing at work.

### 1.7 Flourishing at work as a desired work functioning

Within the SE based on CA framework, functionings refer to individuals’ realized states of being and doing, whereas capabilities represent their opportunities to achieve these states (Robeyns, 2017; Sen, 1999). Flourishing at work can therefore be understood as a desired work functioning; that is, a positive state that emerges when employees are able to realize valued work outcomes (Rothmann et al., 2026b).

Flourishing at work reflects high levels of well-being and positive functioning within the work context (Rothmann, 2013; Rothmann et al., 2019). Drawing on Keyes’s (2002, 2007, 2024) conceptualization of mental health, flourishing encompasses both hedonic and eudaimonic well-being and is reflected in emotional, psychological, and social well-being. In the workplace, emotional well-being refers to positive affect and satisfaction with one’s work, psychological well-being reflects experiences such as competence, autonomy, engagement, meaning, and growth, and social well-being reflects employees’ sense of belonging, contribution, and positive relationships within their work environments (Rautenbach and Rothmann, 2017). Although flourishing reflects high levels of well-being, employees may still experience negative affect, such as frustration, worry, or stress, particularly when workplace demands exceed available resources (Rautenbach and Rothmann, 2017; Rojas and Veenhoven, 2013).

Within the SE based on CA framework, employees who are able and enabled to achieve what they value in their work are more likely to experience positive emotions and satisfaction, meaning and engagement, and positive relationships and contribution within their work environments (Rautenbach and Rothmann, 2017). Flourishing therefore represents one of the key forms of functioning that SE seeks to promote (Rothmann et al., 2026b).

Empirical evidence supports the relationship between work capabilities and flourishing at work. Broader capability sets have been associated with higher levels of emotional, psychological, and social well-being, whereas resource-constrained environments may undermine flourishing by limiting employees’ opportunities to achieve valued work outcomes (Barnard et al., 2023c; Murangi et al., 2022). Similar findings have emerged from flourishing research more broadly, where job satisfaction, work engagement, and job resources have been linked to higher well-being and lower negative affect (Diedericks and Rothmann, 2013; Janse Van Rensburg et al., 2018). Employees who flourish at work also tend to demonstrate stronger in-role and extra-role performance (Redelinghuys et al., 2019). Collectively, these findings suggest that when employees possess opportunities to realize valued work outcomes, they are more likely to experience sustained well-being and positive functioning at work (Gürbüz et al., 2023; Rothmann et al., 2026b).

Understanding these relationships is particularly important among EMS personnel, whose work environments are characterized by persistent job demands, trauma exposure, and resource constraints. Such conditions may restrict employees’ opportunities to realize valued work outcomes and consequently limit their capacity to flourish at work (Ericsson et al., 2022; Stassen et al., 2013; Wijk et al., 2020). Understanding flourishing among EMS personnel therefore requires examining how job demands and job resources shape their work capabilities and, ultimately, their well-being at work.

## 2 Current study

The SE based on CA model proposes that employees’ well-being and long-term functioning depend not only on the resources available to them, but also on their opportunities to convert these resources into valued work outcomes (Sen, 2009; Van der Klink et al., 2016; Van der Klink and Rothmann, 2026). From this perspective, job demands and job resources are important because they shape employees’ opportunities to develop and realize work capabilities, which may ultimately influence their well-being at work.

These processes are particularly relevant in the EMS context. EMS personnel operate in environments characterized by high emotional demands, substantial work pressure, exposure to traumatic events, and the need to make rapid life-saving decisions (Cunningham et al., 2022; Mishra et al., 2010; Van Puyvelde et al., 2023). Although job resources such as autonomy at work and co-worker support may help employees manage these challenges (Bakker et al., 2005; Demerouti and Bakker, 2011; Tummers and Bakker, 2021), the extent to which such resources translate into opportunities to achieve valued work outcomes remains unclear.

While the JD-R model has been widely used to explain employee well-being and functioning (Lesener et al., 2019; Schaufeli, 2017), relatively little research has examined how job demands and job resources are associated with employees’ work capability sets and how these capabilities relate to flourishing at work, particularly among EMS personnel working in South Africa, a non-WEIRD (Western, Educated, Industrialized, Rich, and Democratic) context.

Accordingly, the present study adopted a person-centered approach to examine the work capability sets of EMS personnel in South Africa. Specifically, the study sought to identify distinct capability sets and investigate how these sets differed regarding job demands (emotional demands and work pressure), job resources (autonomy at work and co-worker support), flourishing at work (emotional, psychological, and social well-being), and negative affect.

The following hypotheses were formulated:

*Hypothesis 1 (H1):* EMS personnel’s emotional demands (H1a) and work pressure (H1b) are negatively associated with their work capabilities.

*Hypothesis 2 (H2):* EMS personnel’s autonomy at work (H2a) and co-worker support (H2b) are positively associated with their work capabilities.

*Hypothesis 3 (H3):* EMS personnel’s work capabilities are positively associated with their flourishing at work (H3a) and negatively associated with negative affect (H3b).

*Hypothesis 4 (H4):* Distinct latent capability sets will emerge among EMS personnel in a South African context.

*Hypothesis 5 (H5):* EMS personnel’s latent capability sets differ regarding their job demands (H5a), job resources (H5b), flourishing at work (H5c), and negative affect (H5d).

## 3 Materials and methods

### 3.1 Research design

This study employed a quantitative research design to examine the relationships among EMS personnel’s job demands, job resources, work capabilities, and flourishing at work. Data were collected using pre-existing validated measurement instruments, enabling the statistical analysis of numerical data. Quantitative research designs are well suited for examining relationships among variables and evaluating theoretical propositions using standardized measures (Creswell and Creswell, 2020).

A cross-sectional survey design was used to collect data from EMS personnel at a single point in time. Such designs are widely applied in organizational research to investigate relationships among work characteristics and employee outcomes and to identify factors that may inform future theory development and workplace interventions (Spector, 2019). The design was considered appropriate because the relationships among job demands, job resources, work capabilities, and flourishing at work remain relatively underexplored among EMS personnel, particularly within the South African context.

The study incorporated both variable-centered and person-centered analytical approaches to examine complementary aspects of work capability realization. In the variable-centered analyses, the seven work capabilities were treated as indicators of a common latent construct to examine their shared variance and overall associations with job demands, job resources, and flourishing at work. Additionally, the associations between each of the seven individual work capabilities and the other study variables were examined to provide a more differentiated understanding of these relationships. In contrast, the person-centered approach, using latent class analysis, focused on differences in the configurations of the seven individual work capabilities. This approach enabled the identification of distinct capability sets among EMS personnel and the examination of differences between these subgroups regarding their job demands, job resources, flourishing at work, and negative affect. These approaches provided complementary perspectives on the overall realization, individual associations, and configurations of work capabilities among EMS personnel.

### 3.2 Participants

The study population comprised operational EMS personnel working in South Africa, including Basic Life Support, Intermediate Life Support, and Advanced Life Support practitioners (Health Professions Council of South Africa, 2026). Participants were recruited from across the South African EMS sector. The sample’s demographic characteristics are presented in Table 1.

**Table 1:** Demographic characteristics of participants (*N* = 197).

Demographic variable	Grouping	*n*	%
Gender	Female	71	36.0
	Male	125	63.5
	Missing	1	0.5
Ethnicity	African	73	37.1
	Coloured	21	10.7
	White	100	50.8
	Prefer not to say	3	1.5
Language	English	67	34.0
	Afrikaans	59	29.9
	Sepedi	10	5.1
	Sesotho	16	8.1
	Tshivenda	3	1.5
	isiXhosa	12	6.1
	isiZulu	12	6.1
	siSwati	1	0.5
	Tswana	16	8.1
	Xitsonga	1	0.5
Highest educational level	National senior certificate	57	28.9
	Higher certificate	56	28.4
	National diploma	22	11.2
	Bachelor’s degree	25	12.7
	Honour’s degree	18	9.1
	Master’s degree	10	5.1
	Doctoral degree	1	0.5
	Missing	8	4.1
Contract type	Full-time	178	90.4
	Part-time	10	5.1
	Temporary	6	3.0
	Other	3	1.5
Province	Gauteng	96	48.7
	Free State	7	3.6
	Mpumalanga	10	5.1
	Limpopo	5	2.5
	North West	29	14.7
	Kwa-Zulu Natal	7	3.6
	Eastern Cape	8	4.1
	Western Cape	29	14.7

All ethnicity descriptors are used in line with the terminology of the Employment Equity Act 55 of 1998, which provides for designated and non-designated groups. For example, Coloured is an official term in South Africa used to refer to citizens of mixed ethnic origins. These terms are used for legislative and reporting purposes only, and no offence is intended.

As shown in Table 1, most participants were male (63.5%), White (50.8%), and identified English as their first language (34.0%). The highest level of education reported by most participants was a National Senior Certificate (28.9%) or Higher Certificate (28.4%). The majority were employed on a full-time basis (90.4%) and worked in the Gauteng Province (48.7%). The sample included EMS personnel across different levels of professional registration and represented a broad range of demographic and employment characteristics.

### 3.3 Measuring instruments

A biographical questionnaire was used to collect demographic information, including participants’ ethnicity, gender, age, home language, highest educational qualification, employment type, years of employment, and province of employment.

The Job Demands-Resources Questionnaire (JDRQ) (Bakker, 2014) was used to assess EMS personnel’s job demands and resources. Two job demands and two job resources were assessed using 16 items: *emotional demands* (six items; e.g., “Is your work emotionally demanding?”), *work pressure* (four items; e.g., “Do you have to work at speed?”), *autonomy at work* (three items; e.g., “Do you have control over how your work is carried out?”), and *co-worker support* (three items; e.g., “If necessary, can you ask your colleagues for help?”). Responses were recorded on a five-point scale ranging from 1 (never) to 5 (very often). In the present study, the internal consistency of the subscales was acceptable, with McDonald’s omega (ω) coefficients of 0.85 for emotional demands, 0.77 for work pressure, 0.79 for autonomy, and 0.83 for co-worker support. These reliability estimates are consistent with previous studies that also reported acceptable reliability for these JDRQ subscales (Dos Santos Tome and Van Der Vaart, 2020; Rothmann and Buys, 2011).

The Capability Set for Work Questionnaire (CSWQ) (Abma et al., 2016) was used to examine EMS personnel’s work capabilities. The CSWQ includes 21 items measuring seven work values: (1) using knowledge and skills, (2) developing knowledge and skills, (3) involvement in important decisions, (4) building and maintaining meaningful workplace relationships, (5) setting one’s own goals, (6) earning a good income, and (7) contributing to something valuable. Each work value is evaluated in terms of *importance* (e.g., “How important is it to you to be able to develop your knowledge and skills at work?”), *enablement* (e.g., “Does your work offer you enough opportunities to do that?”), and *achievement* (e.g., “To what extent do you succeed in doing so?”). Participants responded on a five-point scale ranging from 1 (not at all) to 5 (very much). The scale demonstrated excellent reliability in the present study (ω = 0.92), which aligns with previous research reporting acceptable reliability for the instrument (Barnard et al., 2023a; De Wet and Rothmann, 2022; Gürbüz et al., 2023; Ragadu and Rothmann, 2023).

The Flourishing at Work Scale-Short Form (FAWS-SF) (Rautenbach and Rothmann, 2017) was used to measure EMS personnel’s flourishing at work and negative affect. The FAWS-SF consists of 21 items assessing three dimensions of flourishing at work, namely (1) *emotional well-being* (four items; e.g., “During the past month at work, how often did you feel happy?”), (2) *psychological well-being* (nine items; e.g., “During the past month at work, how often did you feel that your work activities were meaningful?”), and (3) *social well-being* (five items; e.g., “During the past month at work, how often did you feel that you belonged at your organization?”). Negative affect was assessed separately using three items (e.g., “During the past month at work, how often did you feel upset?”). Participants were requested to respond to the items using a six-point scale ranging from 1 (never) to 6 (every day). In the present study, the subscales showed acceptable internal consistency reliability values (ω = 0.88 for emotional well-being, ω = 0.91 for psychological well-being, ω = 0.90 for social well-being, and ω = 0.77 for negative affect). These reliability estimates are comparable to those reported in previous studies using the FAWS-SF (Ragadu and Rothmann, 2023; Rautenbach and Rothmann, 2017; Redelinghuys et al., 2019).

### 3.4 Research procedure

Scientific approval for the study was obtained from the Optentia Research Unit Scientific Committee and the School of Industrial Psychology and Human Resource Management at the North-West University. Thereafter, ethical clearance was granted by the Economic and Management Sciences Research Ethics Committee (EMS-REC) prior to data collection (NWU-00741-24-A4). Data were collected between July 2024 and October 2025.

Participants were recruited using non-probability convenience and snowball sampling methods (Struwig and Stead, 2013). Initial access was obtained through a private EMS provider. Following organizational approval, a representative from the organization’s research department distributed the study information, informed consent form, and survey link to EMS personnel. Participants were encouraged to share the survey with colleagues who met the inclusion criteria, thereby facilitating recruitment through professional networks.

To broaden participation, additional EMS personnel were recruited through independent EMS providers across South Africa. An EMS professional also assisted with recruitment by distributing the study invitation through professional communication channels, including WhatsApp broadcast groups and email networks commonly used within the EMS community.

Data were collected primarily through an online survey hosted on the QuestionPro platform, enabling participation from EMS personnel across multiple provinces. To enhance accessibility, paper-based questionnaires were also administered. With permission from participating organizations, the researcher visited operational EMS branches in the Gauteng and North West provinces, where questionnaires were distributed and collected upon completion. Gauteng was specifically targeted for in-person data collection because it contains approximately 30% of South Africa’s EMS workforce (Tiwari et al., 2021).

Participation was voluntary and anonymous. Only individuals currently employed in operational EMS roles and able to provide informed consent were eligible to participate. For the online survey, informed consent was obtained electronically before participants could access the questionnaire. For paper-based surveys, information regarding the purpose of the study, voluntary participation, and anonymity was provided on the introductory page of the questionnaire.

No identifying information was collected, and all data were stored in accordance with the requirements of the Protection of Personal Information Act (POPIA). All instruments were administered in English, the operational language commonly used within EMS in South Africa.

### 3.5 Data analysis

Data were analyzed using SPSS Version 30 (IBM Corporation, 2020), Mplus Version 9 (Muthén and Muthén, 2017), and Jamovi Version 2.6.26 (The jamovi project, 2024). Descriptive statistics were calculated to describe the characteristics of the participants and the distribution of the study variables. The reliability of the measurement scales was assessed using Cronbach’s alpha (α) and McDonald’s omega (ω) coefficients, with values of 0.70 or higher considered acceptable (Field, 2013; Nunnally and Bernstein, 1994). For work capabilities, reliability was assessed using the original CSWQ items before dichotomization. Both variable-centered and person-centered analytical approaches were applied to provide complementary insights into the associations among job demands, job resources, work capabilities, flourishing at work, and negative affect among EMS personnel in South Africa.

To determine the prevalence of realized work capabilities, responses to the CSWQ were dichotomized for each of the three components (importance, enablement, and achievement). Responses of 4 or 5 were classified as affirmative (1), whereas responses of 1 to 3 were classified as non-affirmative (0). This threshold distinguished positive endorsement from neutral or lower ratings. Consistent with the Capability Model for Work (Van der Klink et al., 2026), a work value was classified as a realized work capability (Capable = 1) only when all three components were affirmatively endorsed. If any of the three components received a non-affirmative response, the work value was classified as not constituting a realized work capability (Capable = 0). This operationalization is consistent with previous applications of the instrument (Barnard et al., 2023a; Murangi et al., 2022; Ragadu and Rothmann, 2023). Frequency analyses were subsequently conducted to determine the proportion of EMS personnel who regarded each work value as important, perceived sufficient opportunities to achieve it, actually achieved it, and met all three conditions required for it to constitute a realized work capability. The resulting seven dichotomous work capability indicators were used in both the confirmatory factor analysis (CFA) and latent class analysis (LCA).

CFA was conducted to evaluate the measurement model of job demands, job resources, work capabilities, flourishing at work, and negative affect (Geiser, 2012). The seven dichotomous work capability indicators were specified as indicators of an overall latent work capability factor. Flourishing at work was represented by three distinct latent factors, namely emotional, psychological, and social well-being, while negative affect was specified as a separate latent factor. The CFA was estimated using the weighted least squares mean and variance adjusted (WLSMV) estimator, which is appropriate for models involving categorical or ordinal indicators. Model fit was assessed using multiple goodness-of-fit indices, including the chi-square statistic (*χ*^2^; absolute model fit), standardized root mean square residual (SRMR), root mean square error of approximation (RMSEA), comparative fit index (CFI), and Tucker-Lewis index (TLI) (West et al., 2012). Values of CFI and TLI above 0.90 indicated acceptable fit and above 0.95 excellent fit. RMSEA and SRMR values below 0.08 were considered indicative of acceptable fit, with RMSEA reported together with its 90% confidence interval (Hu and Bentler, 1999; Wang and Wang, 2020). Standardized factor loadings were also examined, with values of 0.30 generally considered acceptable and values of 0.40 or higher preferred, provided that the loadings were statistically significant (*p* < 0.05) (Wang and Wang, 2020).

Given the sample size (*N* = 197) and the complexity of the measurement model, the CFA findings were interpreted with consideration of the number of indicators and latent constructs, the estimation method, model fit, and the magnitude of the standardized factor loadings. Although the WLSMV estimator is appropriate for ordinal and categorical indicators, its use does not eliminate potential concerns regarding parameter stability in relatively small samples. Accordingly, acceptable model fit and factor loadings were considered supportive of the specified measurement model, but not conclusive evidence of sample size adequacy.

Correlation analyses were conducted to examine associations among the study variables using factor scores derived from the measurement model. Specifically, factor scores for the overall work capability construct, derived from the seven dichotomous capability indicators, were used to examine associations with job demands, job resources, flourishing at work, and negative affect. Additional correlation analyses were conducted to examine associations between each of the seven individual dichotomous work capability indicators and these same variables, using point-biserial correlations (Barnard et al., 2023b). Statistical significance was evaluated at *p* < 0.05. The standard criteria for correlation effect sizes were used, with absolute correlation coefficients of 0.10, 0.30, and 0.50 indicating small, medium, and large effects, respectively (De Beer et al., 2022).

To identify distinct work capability sets, LCA was conducted using maximum likelihood estimation with robust standard errors (MLR). Whereas the CFA examined the shared variance among the seven dichotomous work capability indicators, the LCA identified subgroups of EMS personnel with similar configurations of realized work capabilities. Competing models with increasing numbers of classes were compared using the Akaike information criterion (AIC), Bayesian information criterion (BIC), and sample-size adjusted BIC (aBIC), with lower values indicating better model fit (Kline, 2011; Wang and Wang, 2020). The Lo–Mendell–Rubin likelihood ratio test (LMR-LR), adjusted Lo–Mendell–Rubin test (aLMR), and bootstrapped likelihood ratio test (BLRT) were also used to determine whether models with additional classes significantly improved model fit (Lo et al., 2001). Classification accuracy was evaluated using entropy values, with values closer to 1.00 (preferably ≥ 0.80) indicating better classification quality. Average posterior class probabilities were also inspected to assess classification precision, with values above 0.80 considered acceptable (Geiser, 2012). The final class solution was selected by considering statistical fit indices, classification quality, and substantive interpretability of the identified capability sets.

Finally, the Bolck-Croon-Hagenaars (BCH) approach (Bolck et al., 2004) was used to examine differences between the identified work capability sets in terms of job demands, job resources, flourishing at work, and negative affect.

## 4 Results

### 4.1 Capabilities of EMS personnel

Frequency analyses were conducted to examine the importance, enablement, and achievement of the seven measured work values, as well as the proportion of EMS personnel who met all three conditions for these values to constitute realized work capabilities. Table 2 presents the percentages for each component and the corresponding realized work capabilities.

**Table 2:** Work capabilities of EMS personnel.

Work capability	Important	Enabled	Achieved	Capable
(1) Using knowledge and skills	93.9	61.4	67.0	56.3
(2) Developing knowledge and skills	92.4	46.2	51.8	39.6
(3) Involvement in important decisions	69.5	41.1	41.6	32.5
(4) Meaningful relationships at work	76.1	49.7	50.3	39.6
(5) Setting own goals	80.7	43.1	48.0	39.1
(6) Earning a good income	87.8	34.2	33.2	25.5
(7) Contributing to something valuable	76.5	42.9	40.8	34.7

Percentages represent the proportion of participants who affirmatively endorsed each component. Responses of 4 or 5 were classified as affirmative (yes), whereas responses of 1 to 3 were classified as non-affirmative (no). A work value was classified as a realized work capability (Capable) only when a participant affirmatively endorsed all three components: importance, enablement, and achievement.

As shown in Table 2, most participants regarded the work values as important, with percentages ranging from 69.5% (involvement in important decisions) to 93.9% (using knowledge and skills). The proportion of participants reporting that their work environment enabled them to realize these values ranged from 34.2% (earning a good income) to 61.4% (using knowledge and skills). Achievement levels ranged from 33.2% (earning a good income) to 67.0% (using knowledge and skills).

Based on the proportion of participants who affirmatively endorsed all three components (importance, enablement, and achievement), the most prevalent work capabilities among EMS personnel were using knowledge and skills (56.3%), developing knowledge and skills (39.6%), and building and maintaining meaningful relationships at work (39.6%). In contrast, the least prevalent work capabilities were earning a good income (25.5%), involvement in important decisions (32.5%), and contributing to something valuable (34.7%).

Table 2 therefore provides an overview of EMS personnel’s perceived work capabilities before examining the relationships between job demands, job resources, capability sets, and flourishing at work.

### 4.2 Measurement model of job demands-resources, capabilities, flourishing, and negative affect

CFA was conducted to evaluate the measurement model of job demands, job resources, work capabilities, flourishing at work, and negative affect. Job demands (emotional demands and work pressure), job resources (autonomy at work and co-worker support), and work capabilities were specified as unidimensional latent factors, with their respective items loading on a single factor. Flourishing at work was represented by three distinct latent factors, namely emotional well-being, psychological well-being, and social well-being, while negative affect was specified as a separate latent factor.

The measurement model demonstrated acceptable fit to the data: *χ*^2^ = 1528.29 (*df* = 866; *p* < 0.001); CFI = 0.94; TLI = 0.93; RMSEA = 0.06 (90% CI: 0.06; 0.07); SRMR = 0.076. Although the chi-square statistic was significant, *χ*^2^ is sensitive to sample size and was therefore considered alongside the other fit indices. Factor loadings were acceptable across all constructs. Standardized factor loadings were as follows: emotional demands (λ = 0.71 to 0.86; *M* = 0.78); work pressure (λ = 0.59 to 0.95; M = 0.72); autonomy at work (λ = 0.74 to 0.81; *M* = 0.77); co-worker support (λ = 0.75 to 0.93; *M* = 0.84); work capabilities (λ = 0.78 to 0.93; *M* = 0.85); emotional well-being (λ = 0.76 to 0.92; M = 0.82); psychological well-being (λ = 0.63 to 0.87; M = 0.77); social well-being (λ = 0.76 to 0.90; M = 0.84); negative affect (λ = 0.60 to 0.84; M = 0.76).

Overall, the factor loadings indicated that the items loaded adequately on their intended latent constructs, providing support for the specified measurement model of job demands, job resources, work capabilities, flourishing at work, and negative affect. Factor scores derived from the measurement model were subsequently used to examine associations among the study variables. However, given the relatively modest sample size and complexity of the measurement model, these findings should be interpreted cautiously, as acceptable model fit and factor loadings do not necessarily establish the stability of the estimated parameters or factor scores.

Given the high correlations observed between certain latent constructs (see Table 3), additional analyses were conducted to assess discriminant validity. The original nine-factor measurement model was compared with four alternative eight-factor models in which pairs of highly correlated constructs were combined into single factors. WLSMV-adjusted chi-square difference tests indicated that combining emotional and psychological well-being (Δ*χ*^2^(8) = 28.57, *p* < 0.001), work capabilities and autonomy at work (Δ*χ*^2^(8) = 32.96, *p* < 0.001), work capabilities and co-worker support (Δ*χ*^2^(8) = 50.38, *p* < 0.001), and emotional demands and work pressure (Δ*χ*^2^(8) = 68.16, *p* < 0.001) resulted in significantly poorer model fit compared with the original nine-factor model. These findings provided support for retaining the constructs as distinct latent factors. Nevertheless, the particularly high correlation between emotional and psychological well-being suggests substantial shared variance, which should be considered when interpreting their associations with other variables.

**Table 3:** Descriptive statistics, reliabilities, and correlations of scales.

Variables	ω	α	Mean	SD	1	2	3	4	5	6	7	8
(1) Emotional demands	0.85	0.85	3.37	0.91	–	–	–	–	–	–	–	–
(2) Work pressure	0.77	0.77	3.35	0.94	0.71^***^	–	–	–	–	–	–	–
(3) Autonomy at work	0.79	0.79	3.17	1.03	0.05	0.16^*^	–	–	–	–	–	–
(4) Co-worker support	0.83	0.83	3.03	1.04	−0.01	−0.00	0.63^***^	–	–	–	–	–
(5) Work capabilities	0.92	0.92	3.61	0.65	−0.21^**^	0.08	0.76^***^	0.78^***^	–	–	–	–
(6) Emotional well-being	0.88	0.87	3.91	1.19	−0.26^***^	−0.08	0.47^***^	0.61^***^	0.72^***^	–	–	–
(7) Psychological well-being	0.91	0.91	4.03	1.17	−0.24^***^	−0.03	0.47^***^	0.60^***^	0.72^***^	0.95^***^	–	–
(8) Social well-being	0.90	0.89	3.60	1.40	−0.29^***^	−0.04	0.49^***^	0.63^***^	0.78^***^	0.87^***^	0.89^***^	–
(9) Negative affect	0.77	0.75	2.99	1.13	0.42^***^	0.19^**^	−0.34^***^	−0.45^***^	−0.55^***^	−0.67^***^	−0.60^***^	−0.62^***^

ω, McDonald’s omega coefficient; α, Cronbach’s alpha coefficient; SD, Standard Deviation; ^*^p < 0.05; ^**^p < 0.01; ^***^p ≤ 0.001; 0.10 ≤ *r* < 0.30, small effect; 0.30 ≤ *r* < 0.50, medium effect; *r* ≥ 0.50, large effect. Factor scores saved from the measurement model were used to compute the correlations. For work capabilities, reliability coefficients were calculated using the original CSWQ items before dichotomization, whereas the factor scores were derived from the seven dichotomous work capability indicators.

Common method variance (CMV) was considered because the study relied on self-reported data collected using a single questionnaire. An unmeasured latent method factor approach was initially specified. However, the model produced an improper solution involving a negative residual variance and a non-positive-definite residual covariance matrix. Consequently, the method-factor estimates were not interpreted. As a supplementary diagnostic, Harman’s single-factor test was conducted. The first factor accounted for 35.6% of the variance, which was below the commonly used 50% threshold. However, given the methodological limitations of this test, the result cannot be interpreted as evidence that CMV was absent or negligible. Therefore, the extent to which CMV may have influenced the observed associations remains uncertain.

### 4.3 Descriptive statistics, reliabilities, and correlations of the scales

Table 3 presents the descriptive statistics, reliability coefficients, and correlations among the study variables. The reliability coefficients for all six scales were acceptable, with McDonald’s omega (ω) and Cronbach’s alpha (α) values exceeding 0.70 (Field, 2013; Nunnally and Bernstein, 1994).

Regarding the associations between job demands, job resources, and work capabilities, emotional demands showed a statistically significant negative association with work capabilities (small effect). In contrast, autonomy at work and co-worker support were positively associated with work capabilities, both demonstrating large effects. Work capabilities were positively associated with emotional well-being, psychological well-being, and social well-being, and negatively associated with negative affect, all with large effect sizes.

With regard to job demands and flourishing at work, emotional demands were negatively associated with emotional, psychological, and social well-being (small effects) and positively associated with negative affect (medium effect). Work pressure was not significantly associated with the three well-being components but was positively associated with negative affect (small effect). Regarding job resources and flourishing, both autonomy at work (medium effects) and co-worker support (large effects) were positively associated with emotional, psychological, and social well-being, and negatively associated with negative affect (medium effects).

In addition to the associations involving the overall work capability construct reported in Table 3, point-biserial correlations were conducted to examine the associations between each of the seven individual dichotomous work capability indicators and job demands, job resources, flourishing at work, and negative affect (these associations are not included in Table 3). Emotional demands were negatively associated only with earning a good income (*r* = −0.16; *p* < 0.05). Work pressure was positively associated with involvement in important decisions (*r* = 0.22; *p* < 0.01) and contributing to something valuable (*r* = 0.22; *p* < 0.01).

Regarding job resources, both autonomy at work and co-worker support were positively associated with all seven work capabilities (*p* < 0.001), with medium to large effects. Autonomy at work: using knowledge and skills (*r* = 0.47), developing knowledge and skills (*r* = 0.48), involvement in important decisions (*r* = 0.56), building and maintaining meaningful workplace relationships (*r* = 0.47), setting own goals (*r* = 0.50), earning a good income (*r* = 0.39), and contributing to something valuable (*r* = 0.52). Co-worker support: using knowledge and skills (r = 0.44), developing knowledge and skills (*r* = 0.45), involvement in important decisions (*r* = 0.41), building and maintaining meaningful workplace relationships (*r* = 0.51), setting own goals (*r* = 0.41), earning a good income (*r* = 0.40), and contributing to something valuable (*r* = 0.47).

All work capabilities were statistically significantly (*p* < 0.001) associated with the three flourishing components (i.e., emotional, psychological, and social well-being, respectively): using knowledge and skills (*r* = 0.33; *r* = 0.33; *r* = 0.35), developing knowledge and skills (*r* = 0.36; *r* = 0.37; *r* = 0.39), involvement in important decisions (*r* = 0.34; *r* = 0.34; *r* = 0.40), building and maintaining meaningful workplace relationships (*r* = 0.48; *r* = 0.49; *r* = 0.50), setting own goals (*r* = 0.37; *r* = 0.38; *r* = 0.39), earning a good income (*r* = 0.37; *r* = 0.37; *r* = 0.43), and contributing to something valuable (*r* = 0.38; *r* = 0.39; *r* = 0.44).

Lastly, all seven work capabilities were negatively associated with negative affect (*p* < 0.001): using knowledge and skills (*r* = −0.25), developing knowledge and skills (*r* = −0.25), involvement in important decisions (*r* = −0.26), building and maintaining meaningful workplace relationships (*r* = −0.30), setting own goals (*r* = −0.32), earning a good income (*r* = −0.22), and contributing to something valuable (*r* = −0.30).

Based on these findings, Hypothesis 1 was supported for emotional demands (H_1a_), which showed a statistically significant negative association with work capabilities. However, (H_1b_) was not supported, as work pressure was not significantly associated with overall work capabilities. Although work pressure showed statistically significant positive associations with involvement in important decisions and contributing to something valuable, these associations were contrary to the hypothesized negative direction. Hypothesis 2 was supported for autonomy at work (H2a) and co-worker support (H2b) in their associations with work capabilities. Hypothesis 3 was also supported, as work capabilities were positively associated with flourishing at work (H3a) and negatively associated with negative affect (H3b).

### 4.4 Capability sets of EMS personnel

LCA was conducted using the seven dichotomous work capability indicators to identify distinct capability sets among EMS personnel. Table 4 presents the model fit indices for the competing latent class solutions.

**Table 4:** Comparisons of different capability latent class analysis models.

Classes	AIC	BIC	aBIC	Entropy	LMR LR test *p*-value	aLMR LR test *p*-value	BLRT *p*-value	Smallest class membership
1	1802.07	1825.05	1802.88	–	–	–	–	–
2	1393.31	1442.56	1395.04	0.90	< 0.001	< 0.001	< 0.001	40%
**3**	**1368.43**	**1443.94**	**1371.08**	**0.81**	**0.006**	**0.007**	**< 0.001**	**22%**
4	1368.30	1470.08	1371.87	0.84	0.101	0.107	0.240	8%

AIC, Akaike information criterion; BIC, Bayesian information criterion; aBIC, adjusted Bayesian information criterion; LMR LR, Lo–Mendell–Rubin test; aLMR LR, adjusted Lo–Mendell–Rubin test; BLRT, bootstrapped likelihood ration test. Bold text indicates the class-solution selected.

Table 4 presents the model fit indices for the one, two, three, and four-class solutions. The two-class model demonstrated improved model fit compared to the one-class solution: ΔAIC = −408.76; ΔBIC = −382.49; ΔaBIC = −407.84; LMR LR (*p* < 0.001); aLMR LR (*p* < 0.001); BLRT (*p* < 0.001). The three-class solution showed a further improvement relative to the two-class solution: ΔAIC = −24.88; ΔBIC = 1.38; ΔaBIC = −23.96; LMR LR (*p* = 0.006); aLMR LR (*p* = 0.007); BLRT (*p* < 0.001). However, the BIC increased slightly from 1442.56 for the two-class solution to 1443.94 for the three-class solution (ΔBIC = +1.38), indicating that the BIC criterion favored the two-class solution. Thus, the statistical indices provided mixed evidence regarding the two- versus three-class solutions.

The three-class solution was retained based on a combination of statistical evidence, classification quality, and substantive interpretability. Specifically, the AIC, aBIC, and likelihood ratio tests supported the three-class solution, while the identified classes demonstrated substantively distinct capability profiles and satisfactory classification accuracy (average posterior probabilities = 0.88–0.94). Class 1 consisted of 94 participants (48%), Class 2 included 44 participants (22%), and Class 3 comprised 59 participants (30%), indicating acceptable class proportions (Wang and Wang, 2020). In contrast, the four-class solution did not provide a meaningful improvement in model fit compared to the three-class solution: ΔAIC = −13.0; ΔBIC = 26.14; ΔaBIC = 0.79; LMR LR (*p* = 0.101); aLMR LR (*p* = 0.107); BLRT (*p* = 0.240).

However, the selection of the three-class solution should be interpreted with caution. Tein et al. (2013) demonstrated that the ability to correctly identify latent classes depends not only on sample size but also on class separation, the number of indicators, and the model-selection criteria employed. Their findings particularly emphasized the importance of class separation in determining statistical power, suggesting that sample size alone is insufficient to establish the adequacy of latent class solutions. Although the average posterior class probabilities (0.88–0.94) indicated satisfactory classification precision, these values do not independently establish the stability or replicability of the identified classes. Given the relatively modest sample size (*N* = 197), the smallest class comprising 44 participants (22%), and the conflicting evidence provided by the information criteria, the three-class solution should be regarded as a theoretically interpretable representation of capability heterogeneity within the present sample rather than a definitive classification of EMS personnel. Further research using larger and independent samples is needed to establish the stability and replicability of the identified capability sets.

The three distinct latent EMS personnel work capability sets (or sub-populations of work capability classes) are illustrated in Figure 1.

**Figure 1:**
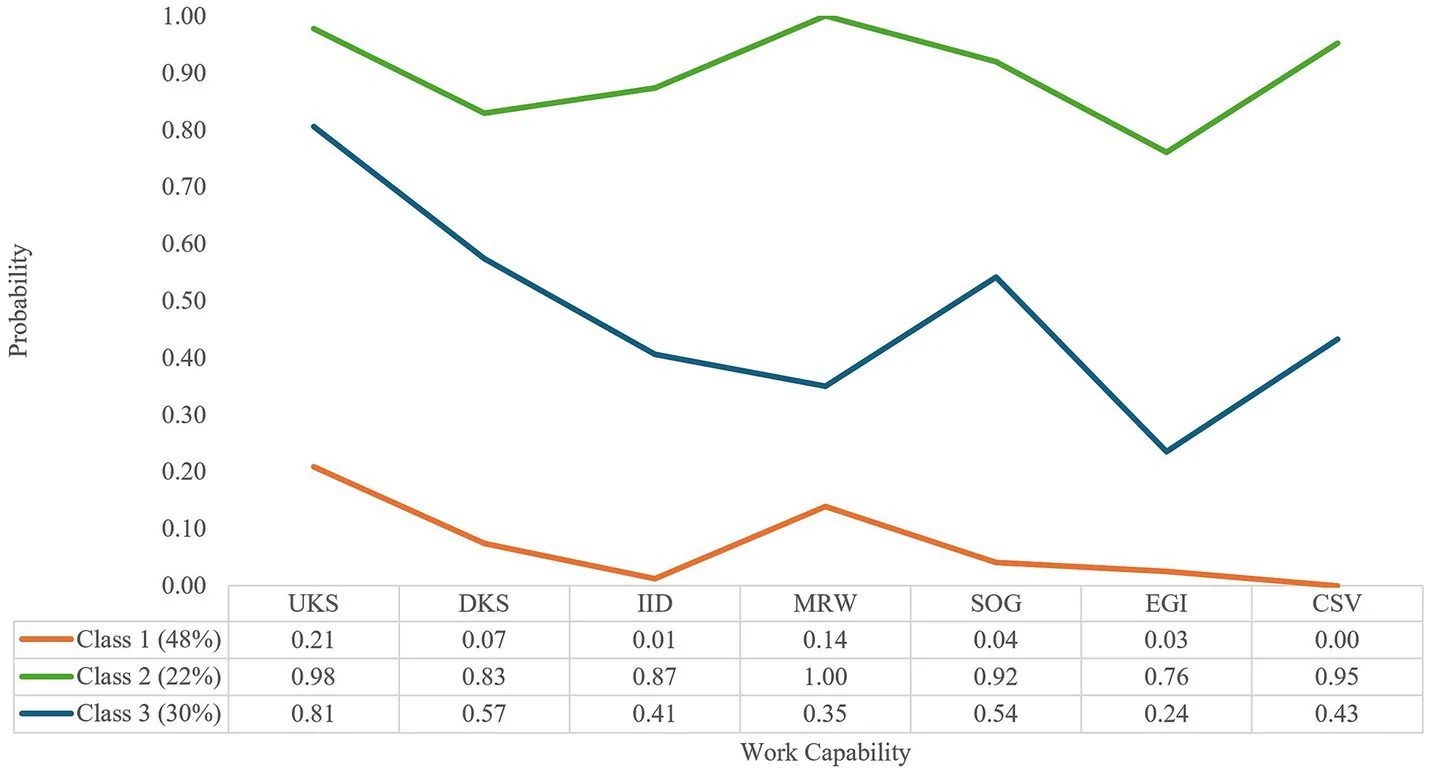
Latent EMS personnel capability sets. UKS, using knowledge and skills; DKS, developing knowledge and skills; IID, involved in important decisions; MRW, meaningful relationships at work; SOG, setting own goals; EGI, earning a good income; CSV, contributing to something valuable; Class 1, weak capability set; Class 2, robust capability set; Class 3, constrained capability set.

The three classes depicted in Figure 1 can be interpreted as follows: (a) Class 1: weak capability set (Barnard et al., 2023a; Mnxuma et al., 2026; Ragadu and Rothmann, 2023). EMS personnel in this group (which accounts for nearly half of the sampled population) reported low levels across all seven work capabilities. Slightly higher scores were observed for using knowledge and skills and building and maintaining meaningful relationships at work, while particularly low scores were evident for involvement in important decisions and contributing to something valuable. (b) Class 2: robust capability set (Barnard et al., 2023a; Mnxuma et al., 2026; Ragadu and Rothmann, 2023). EMS personnel assigned to this class reported high levels across most of the seven work capabilities. The strongest capabilities were building and maintaining meaningful relationships at work, using knowledge and skills, contributing to something valuable, and setting own goals. Although earning a good income showed a slightly lower score than the other work capabilities, it remained relatively high compared to the other capability sets. (c) Class 3: constrained capability set (Mnxuma et al., 2026). EMS personnel in this group demonstrated relatively high scores for using and developing knowledge and skills, while the remaining capabilities were generally moderate. The earning a good income work capability was below moderate, indicating that this aspect was less frequently realized among members of this class.

Based on these findings, Hypothesis 4 (H_4_), which posits the existence of distinct latent work capability sets among EMS personnel in South Africa, is accepted.

### 4.5 Comparisons of EMS personnel’s work capability sets with job demands-resources and work functionings

Table 5 shows the differences in job demands, job resources, and flourishing at work, and negative affect among the three identified latent EMS personnel work capability sets.

**Table 5:** Comparisons of different work capability sets with job demands, job resources, flourishing at work, and negative affect.

Variable	*χ* ^2^	Class	Mean	SE	Class Comparison	*χ* ^2^
Emotional demands	2.49	1	−0.01	0.08	1 vs. 2	1.36
		2	−0.19	0.13	1 vs. 3	0.48
		3	0.09	0.10	2 vs. 3	2.46
Work pressure	4.34	1	−0.11	0.06	1 vs. 2	3.39
		2	0.11	0.10	1 vs. 3	1.80
		3	0.05	0.09	2 vs. 3	0.21
Autonomy at work	113.79^***^	1	−0.42	0.06	1 vs. 2	103.72^***^
		2	0.64	0.09	1 vs. 3	32.22^***^
		3	0.18	0.08	2 vs. 3	13.15^***^
Co-worker support	87.15^***^	1	−0.36	0.06	1 vs. 2	85.39^***^
		2	0.64	0.09	1 vs. 3	17.69^***^
		3	0.09	0.08	2 vs. 3	20.15^***^
Emotional well-being	76.41^***^	1	−0.31	0.08	1 vs. 2	73.29^***^
		2	0.69	0.08	1 vs. 3	4.48^*^
		3	−0.03	0.10	2 vs. 3	28.43^***^
Psychological well-being	72.36^***^	1	−0.32	0.08	1 vs. 2	71.78^***^
		2	0.66	0.09	1 vs. 3	6.23^*^
		3	0.01	0.10	2 vs. 3	21.89^***^
Social well-being	94.72^***^	1	−0.36	0.07	1 vs. 2	93.86^***^
		2	0.69	0.08	1 vs. 3	10.51^**^
		3	0.05	0.09	2 vs. 3	25.35^***^
Negative affect	27.97^***^	1	0.24	0.08	1 vs. 2	27.94^***^
		2	−0.47	0.11	1 vs. 3	3.42
		3	−0.01	0.09	2 vs. 3	9.69^**^

*χ*^2^, chi-square; SE, standard error; ^*^p < 0.05; ^**^p < 0.01; ^***^p < 0.001.

As shown in Table 5, the three identified capability classes did not differ statistically significantly (*p* > 0.05) based on the measured job demands (i.e., emotional demands and work pressure). In contrast, the identified EMS personnel work capability classes differed statistically significantly based on their job resources (i.e., autonomy at work and co-worker support).

EMS personnel reported statistically significant different flourishing at work responses based on their work capability sets, apart from between the weak and constrained capability classes regarding their negative affect.

Figure 2 illustrates how EMS personnel differed in their job demands, job resources, flourishing at work, and negative affect across the identified work capability sets.

**Figure 2:**
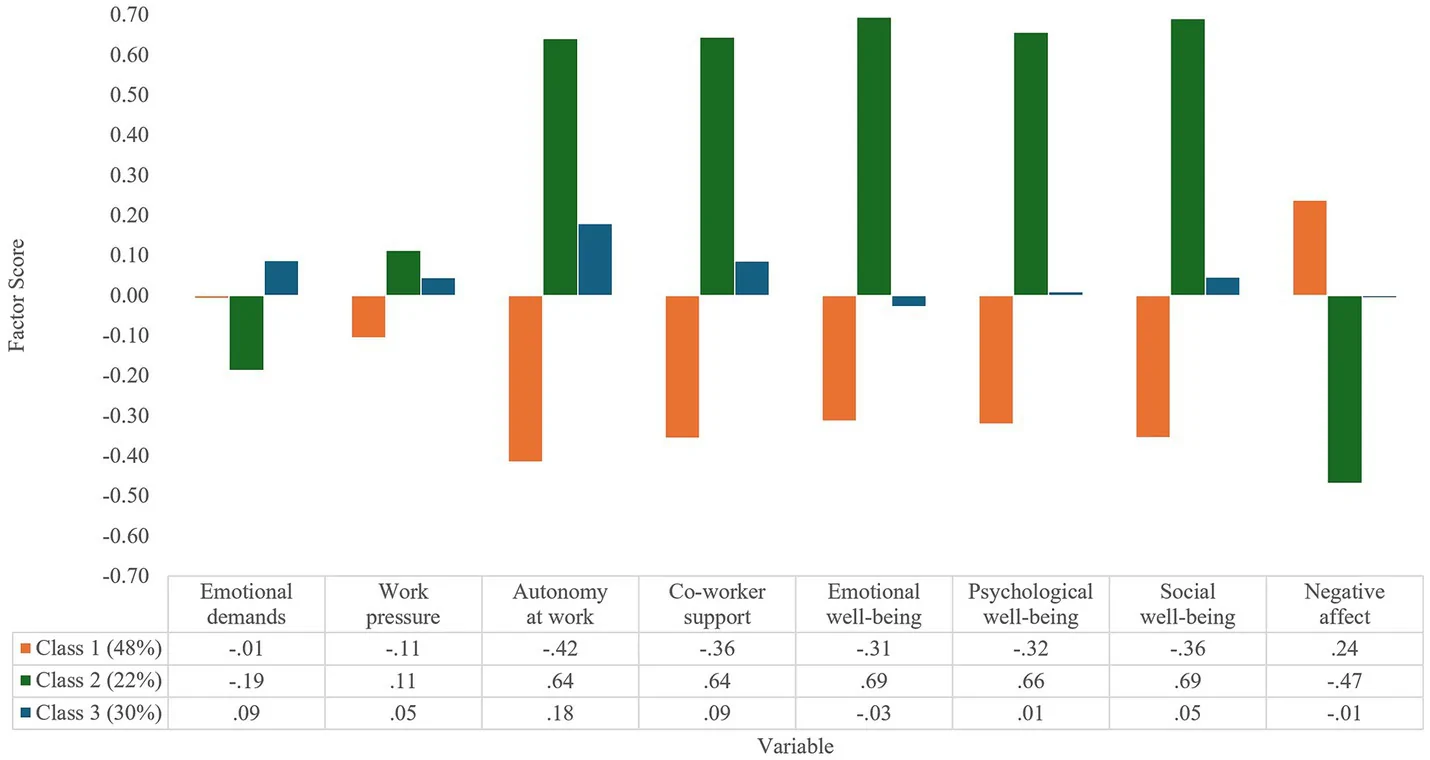
Capability sets, job demands, job resources, flourishing at work, and negative affect of EMS personnel. Class 1, weak capability set; Class 2, robust capability set; Class 3, constrained capability set.

As shown in Figure 2, the measured job demands did not significantly differentiate the capability classes. Overall, the results indicate that the measured job demands could not be clearly attributed to the distinct work capability classes identified among EMS personnel working in South Africa. In contrast, the measured job resources played a significant role in grouping these professionals. EMS personnel in the robust capability set reported noticeably higher levels of autonomy at work and co-worker support compared to those in the weak capability set. While EMS personnel in the constrained capability set did not report levels as high as those in the robust capability set, their scores were still visibly higher than those in the weak capability set. However, the constrained capability set did not differ substantially from the weak capability set in terms of negative affect (*p* = 0.065).

Hypothesis 5 (H5) was therefore supported for job resources (H5b), flourishing at work (H5c), and negative affect (H5d), as evidence indicated that EMS personnel’s identified capability sets differed based on these variables. However, the hypothesis was not supported for job demands (H5a).

## 5 Discussion

This study examined the associations between job demands (emotional demands and work pressure), job resources (autonomy at work and co-worker support), work capabilities, flourishing at work, and negative affect among EMS personnel in a South African context. Grounded in the SE based on the CA model (Abma et al., 2016; Van der Klink et al., 2026), the study sought to understand how work environments enable EMS personnel to achieve valued work outcomes and how these opportunities relate to flourishing at work. Viewed collectively, the findings point to three interrelated insights. First, although EMS personnel generally valued the seven work outcomes, their opportunities to realize these outcomes were considerably more restricted. Second, the work environment appeared to matter not merely because it contained demands and resources, but because these conditions may enable or constrain employees’ opportunities to achieve what they value. Third, the person-centered findings suggest that these opportunities occur as broader capability configurations, with more robust capability sets corresponding with more favorable functioning. These findings illustrate how the JD-R and CA perspectives can complement one another in explaining SE.

The findings showed that most EMS personnel regarded all seven work values as important. However, considerably fewer participants reported being enabled to achieve these values or actually achieving them in practice. In particular, earning a good income, contributing to something valuable, and involvement in important decisions emerged as the least realized work values. Similar patterns have been reported among emergency nurses, industrial psychology and human resource management practitioners, and early childhood development practitioners in South Africa (Barnard et al., 2023a; Mnxuma et al., 2026; Ragadu and Rothmann, 2023). Rather than reflecting differences in what EMS personnel value, this discrepancy points to a gap between valued work outcomes and the genuine opportunities available to realize them. This distinction is central to the CA, which differentiates between the resources available to individuals and their substantive opportunities to achieve outcomes they have reason to value (Robeyns, 2017; Sen, 2009; Van der Klink et al., 2026). The relatively low realization of these work values may therefore reflect conversion gaps that, from the CA perspective, could be associated with organizational and contextual conditions affecting employees’ opportunities to translate available resources into valued work outcomes (Rothmann et al., 2026a).

The occupational context of EMS work may help explain some of these gaps. The low endorsement of contributing to something valuable is somewhat unexpected given the life-saving nature of EMS work. Similar observations have been made among emergency nurses and firefighters (Barnard et al., 2023a; Wallace, 2026). EMS personnel typically provide critical interventions before transferring patients to other healthcare providers and proceeding to the next emergency. Consequently, they seldom witness patients’ longer-term recovery or receive direct feedback regarding outcomes. Limited opportunities to observe the impact of their work or receive recognition may gradually diminish perceptions of making a meaningful contribution and reduce feelings of significance (Wallace, 2026). Similarly, involvement in important decisions may be constrained by the highly regulated nature of EMS work. Clinical protocols and standard operating procedures are designed to protect patient safety and minimize risk, leaving relatively little discretion in many operational decisions (Henderson, 2013). While such structures are necessary, they may inadvertently restrict opportunities for participation in decision-making and reduce employees’ perceptions of influence within the organization. These findings illustrate an important capability distinction in which performing socially valuable work or possessing professional expertise does not necessarily mean that employees experience genuine opportunities to realize all the outcomes they value through that work.

At the same time, the capability picture was not uniformly constrained. The most prominent work capabilities among participants were using knowledge and skills, developing knowledge and skills, and building and maintaining meaningful relationships at work. However, with the exception of using knowledge and skills, these capabilities were realized by fewer than half of the participants. These capabilities may be particularly consequential because opportunities and achievements in one domain can create positive spillovers into others. Rothmann et al. (2026a), drawing on the notion of desired functioning, argue that supportive conditions and achievements in one domain may facilitate functioning across other domains, whereas disadvantages may similarly have broader consequences. Opportunities to use and develop professional expertise, for example, may support participation, contribution, and professional growth, while meaningful workplace relationships may provide access to social resources that facilitate other valued outcomes. SE should therefore not be understood solely in terms of whether individual capabilities are present or absent, but also in terms of how opportunities across valued domains may reinforce or constrain one another.

A second central insight concerns how job demands and resources may shape these opportunities. Integrating the JD-R model with the CA provides a useful framework for interpreting this pattern. Whereas the JD-R model identifies job characteristics that can promote or impair employee functioning, the CA directs attention to whether these conditions expand or restrict employees’ substantive opportunities to achieve valued outcomes. Job resources such as autonomy and social support can accordingly be understood as enabling conditions through which capabilities may be developed and exercised, while job demands can operate as constraints when they restrict valued functioning (Van der Vaart et al., 2026). The present findings are consistent with this complementary theoretical interpretation, while also suggesting that different work characteristics may have different associations with the realization of work capabilities.

Emotional demands were negatively associated with work capabilities and flourishing and positively associated with negative affect. These findings align with previous research among healthcare professionals demonstrating that emotional demands undermine work capabilities and desired work outcomes (Barnard et al., 2023b). Emotional demands require sustained emotional regulation and have consistently been linked to psychological strain, poorer well-being, and increased symptoms of distress (Ahmed et al., 2017; Kim et al., 2019). More recent research among EMS personnel in the Czech Republic found that occupational stress was associated with psychological distress, workplace dissatisfaction, and previous considerations of leaving the profession (Lochmannová et al., 2026). These findings highlight the relevance of occupational conditions not only for psychological functioning but also for broader concerns about workforce retention. For EMS personnel, sustained exposure to emotionally demanding situations may therefore have implications beyond psychological strain, as suggested by its negative association with their perceived opportunities to realize valued aspects of work. From a capability perspective, this pattern is consistent with the theoretical proposition that emotional demands may constrain employees’ opportunities to convert available personal and workplace resources into valued outcomes. However, the cross-sectional findings do not establish whether such a conversion process occurred.

Work pressure showed a different pattern. It was not significantly associated with overall work capabilities or flourishing, although it was positively associated with negative affect. This suggests that experiencing high work pressure does not necessarily diminish EMS personnel’s perceptions of their opportunities to achieve valued work outcomes. One possible explanation is that work pressure may be viewed as a normative characteristic of EMS work. Previous research has shown that EMS personnel often normalize challenging work experiences, including exposure to violence and other occupational hazards (Maguire et al., 2018). The positive associations between work pressure and the capabilities of involvement in important decisions and contributing to something valuable may also reflect the rapid decision-making and professional judgement required under high-pressure conditions. However, these interpretations should be treated cautiously because work pressure was not significantly associated with overall work capabilities or flourishing. More broadly, the contrast between emotional demands and work pressure indicates that job demands should not automatically be treated as equivalent capability constraints; their implications may depend on the nature of the demand and how it is experienced within a particular occupational context.

Job resources emerged as particularly important. Both autonomy at work and co-worker support showed strong positive associations with work capabilities and flourishing. Previous research among EMS professionals has similarly highlighted the relevance of job demands and resources for occupational well-being, with burnout differing substantially across demand-resource conditions (Crowe et al., 2020). Furthermore, a systematic review of prehospital EMS research identified supervisor support as an important predictor of job satisfaction, highlighting the broader relevance of workplace support for positive occupational functioning (Thielmann et al., 2023). Although supervisor support differs from the co-worker support examined in the present study, these findings collectively reinforce the importance of considering relational and organizational resources when examining EMS personnel’s functioning. EMS personnel who experienced greater control over their work and stronger support from colleagues reported greater opportunities to realize valued work outcomes and higher levels of well-being. Their relevance may extend beyond their conventional role as resources that facilitate goal attainment or buffer demands. From a capability perspective, resources are valuable insofar as they contribute to what individuals are genuinely able to do and become (Rothmann et al., 2026a; Van der Vaart et al., 2026). The positive associations observed in the present study are consistent with the possibility that autonomy may expand opportunities for professional judgement, choice, self-direction, and participation, while co-worker support may provide relational and social conditions through which EMS personnel can navigate demands and pursue valued work outcomes. However, these proposed enabling processes were not directly examined and should be interpreted as theoretical explanations rather than established mechanisms. This interpretation is consistent with previous research where autonomy may function as a “super capability” that enables employees to convert challenges and demands into opportunities for growth and achievement (Barnard et al., 2023b). Likewise, co-worker support may facilitate capability development by helping employees realize valued work outcomes and navigate the challenges inherent in EMS work (Barnard et al., 2023b; Ragadu and Rothmann, 2026). The findings therefore are consistent with the proposition that the relevance of job resources for SE may lie not only in their presence, but also in the opportunities they may help employees realize.

The person-centered analyses provided further support for this interpretation. Three distinct capability sets emerged: weak, constrained, and robust. Nearly half of participants belonged to the weak capability set, characterized by low endorsement across all capabilities. In contrast, participants in the robust capability set reported high endorsement of most capabilities, while those in the constrained capability set displayed an intermediate profile characterized by strengths in some capabilities and limitations in others. Importantly, these patterns indicate that employees working within the same broadly demanding occupational context may nevertheless experience markedly different configurations of substantive opportunities. However, given the non-probability sampling approach and the geographical and demographic composition of the sample, these capability sets should be interpreted as sample-specific patterns rather than nationally representative profiles of South African EMS personnel.

Job resources rather than job demands differentiated these capability sets. EMS personnel in the robust capability set reported significantly higher levels of autonomy and co-worker support than those in the weak capability set, with the constrained capability set occupying an intermediate position. Capability sets were also strongly associated with flourishing. Participants in the weak capability set reported substantially lower levels of emotional, psychological, and social well-being and higher levels of negative affect than those in the other capability groups. In contrast, participants in the robust capability set reported the highest levels of well-being and the lowest levels of negative affect. These findings align with previous research demonstrating that broader capability sets are associated with greater flourishing and SE (De Wet and Rothmann, 2022; Gürbüz et al., 2023; Ragadu and Rothmann, 2023). Taken together, the person-centered findings suggest that SE may be better understood in terms of configurations of substantive opportunities rather than isolated capabilities. They further support the CA distinction between capabilities as opportunities and functionings as realized states of being and doing in which employees with broader opportunities to achieve what they value also reported more favourable functioning in the form of greater flourishing and lower negative affect.

Collectively, the variable- and person-centered findings provide a more integrated account of SE among EMS personnel. They suggest that understanding employee functioning requires moving beyond the presence of demands and resources alone to consider whether the work environment provides genuine opportunities to achieve valued outcomes. The findings further indicate that enabling resources may be particularly relevant to the development of broader capability sets, while specific demands, such as emotional demands, may constrain both capabilities and well-being. In this sense, integrating the JD-R model with the CA shifts attention from what the work environment provides or requires to what employees are genuinely able to achieve within that environment. From an integrated JD-R and CA perspective, the findings are consistent with the proposition that SE may be understood through the interplay between occupational conditions, employees’ substantive opportunities, and the valued functionings associated with flourishing at work.

## 6 Theoretical and practical implications

This study contributes to the SE literature by clarifying how work conditions, capabilities, and flourishing may be understood in relation to one another. First, the findings strengthen the integration of the JD-R model with the SE model grounded in the CA. The JD-R model identifies job demands and resources that shape employee functioning, whereas the CA directs attention to whether these work conditions translate into substantive opportunities to achieve valued outcomes (Van der Vaart et al., 2026). The present findings suggest that this distinction is consequential because autonomy and co-worker support were associated with broader work capabilities and differentiated capability sets, whereas job demands showed less uniform relationships with capabilities. Integrating the two perspectives therefore extends attention beyond the availability of workplace resources and exposure to demands towards the opportunities that employees are genuinely able to realize within those conditions. Importantly, this perspective suggests that the relevance of job characteristics for SE cannot be fully understood without considering employees’ perceived opportunities to achieve valued work outcomes, although the processes through which these opportunities develop require further investigation.

Second, the findings refine our understanding of how job demands may relate to SE. Emotional demands were associated with more restricted work capabilities and poorer flourishing, whereas work pressure was not associated with overall work capabilities or flourishing. This suggests that job demands should not necessarily be conceptualized as uniformly constraining from a capability perspective. Rather, their relevance for SE may depend on whether and how particular demands restrict employees’ substantive opportunities to achieve valued work outcomes. The contrasting associations also suggest that the implications of job demands for capabilities may differ from their implications for psychological functioning, as work pressure was positively associated with negative affect despite its non-significant association with overall work capabilities. This distinction provides a basis for examining the conditions under which specific job demands become capability constraints in future research.

Third, the person-centered findings reinforce the importance of conceptualizing SE in terms of capability sets rather than isolated opportunities. The emergence of weak, constrained, and robust capability sets indicates that employees within the same occupational context may differ substantially in the breadth and configuration of opportunities they report being able to realize. Moreover, the association of these capability sets with flourishing and negative affect is consistent with the distinction between capabilities as substantive opportunities and functionings as realized states of being and doing (Rothmann et al., 2026b; Sen, 2009). Although flourishing and negative affect do not represent direct measures of all valued functionings, their associations with capability sets highlight the relevance of examining opportunities and well-being together. SE may therefore be better understood by considering the configuration of opportunities available to employees and how these configurations relate to valued functioning.

Finally, by focusing on EMS personnel in South Africa, the study contributes to the limited body of research on SE within emergency service professions and non-WEIRD contexts. Work capabilities are inherently sensitive to occupational and contextual conditions because demands, resources, institutional arrangements, and conversion factors may influence what employees are genuinely able to achieve (Rothmann et al., 2026a). Examining capability sets among EMS personnel therefore demonstrates how the SE model grounded in the CA can be applied within a highly demanding emergency healthcare occupation in a non-WEIRD context. However, the identified capability configurations should be understood within the characteristics of the present sample rather than assumed to represent all South African EMS personnel.

The findings also have important practical implications for EMS organizations. Rather than focusing solely on reducing demands or providing resources, organizations should consider whether their work environments enable personnel to realize the work outcomes they value. The discrepancy between the importance attached to several work values and employees’ opportunities to realize them suggests that interventions could begin by identifying where such capability gaps occur and which organizational conditions may be restricting their realization. This could involve periodically assessing the importance, perceived enablement, and achievement of valued work outcomes to identify areas in which organizational improvements may be needed.

The findings particularly highlight autonomy and co-worker support as potentially important enabling conditions. Increasing autonomy may expand EMS personnel’s opportunities to exercise professional judgement and maintain greater control over how they perform their work (Safi-Keykaleh et al., 2020). Within the operational and safety requirements of EMS work, organizations could identify areas in which personnel can exercise appropriate professional discretion and participate in decisions that affect their work. Opportunities for involvement in operational planning, structured consultation, and input into work processes may strengthen employees’ sense of agency without compromising necessary clinical protocols. Such initiatives should focus on opportunities for meaningful participation and professional discretion within established clinical and operational boundaries, rather than assuming that greater autonomy is appropriate in all aspects of emergency care.

Co-worker support could similarly be strengthened through structured peer-support initiatives, team-based reflection sessions, and debriefing practices following particularly demanding incidents (Huang et al., 2022; Mildenhall, 2012). Such practices may not only assist personnel in managing demanding experiences but also strengthen the relational conditions through which valued work outcomes can be realized. Their implementation should nevertheless be sensitive to personnel’s needs and preferences, with access to appropriate professional support where necessary.

Interventions should nevertheless not focus on resources at the expense of managing harmful demands. Emotional demands were associated with more restricted capabilities, lower flourishing, and greater negative affect, indicating the importance of organizational practices that help EMS personnel manage the emotional consequences of emergency work. Strengthening supportive team environments, access to appropriate support mechanisms, and opportunities to process emotionally demanding experiences may therefore complement efforts to expand employees’ work capabilities. Given the cross-sectional nature of the findings, these approaches should be regarded as theoretically informed recommendations rather than interventions whose effectiveness was established by the present study.

Finally, organizations could pay greater attention to capabilities that were particularly difficult for EMS personnel to realize. Where the structure of emergency care limits opportunities to witness patient recovery, mechanisms for communicating patient outcomes, acknowledging successful interventions, and recognizing personnel’s contribution may help make the value of their work more visible (Wallace, 2026). Where feasible and consistent with patient confidentiality requirements, feedback about patient outcomes could provide personnel with a clearer sense of the contribution of their work. Similarly, creating meaningful channels through which EMS personnel can contribute to organizational and operational decisions may expand opportunities for involvement and influence. Given that earning a good income was also among the least realized capabilities, organizations and relevant decision-makers should consider how remuneration structures and opportunities for financial recognition relate to personnel’s valued work outcomes, while recognizing the broader financial and institutional constraints affecting EMS services. Collectively, these practices shift the focus from simply providing resources towards creating work environments in which EMS personnel have genuine opportunities to achieve what they value, flourish at work, and sustain their functioning over time.

## 7 Limitations and recommendations for future research

Several limitations should be considered when interpreting this study’s findings. First, the study used convenience and snowball sampling, with a substantial proportion of participants drawn from Gauteng Province, South Africa. The sample’s demographic composition should also be considered, as 50.8% of participants identified as White and the sample was concentrated in only two provinces. The sample’s racial composition may therefore not fully reflect the national demographic composition of EMS personnel. Although the findings are consistent with previous research conducted in other non-WEIRD contexts (Barnard et al., 2023a; De Wet and Rothmann, 2022; Mnxuma et al., 2026; Murangi et al., 2022; Ragadu and Rothmann, 2023), caution should be exercised when generalizing the results to EMS personnel in other geographical or cultural contexts. Importantly, the three capability classes identified in this study should be interpreted as patterns observed within the present sample rather than as established profiles of South African EMS personnel more broadly. Their prevalence and composition may differ across geographical, demographic, and organizational contexts. Future research should seek to include more geographically and demographically diverse samples and examine whether similar capability patterns emerge across different regions and emergency service settings.

A further limitation concerns the language in which the study instruments were administered. All instruments were administered in English, although only 34% of participants reported English as their first language. Although English is widely used in South African professional and educational contexts, administering the instruments only in English may have affected item comprehension and the extent to which the measures were culturally and linguistically equivalent across participants. This may introduce measurement bias, particularly if participants interpreted specific items differently. Future research should use validated multilingual versions of the instruments, where available, and assess measurement equivalence across language groups. Data were collected using both online and paper-based questionnaires. Although this approach facilitated data collection across participants, differences in administration mode may have influenced how participants responded to the instruments and may have introduced method variance. Future research should test measurement invariance across data collection modes and, where appropriate, conduct sensitivity analyses to determine whether the mode of administration influences the findings.

The sample size should also be considered when interpreting the results as it may have implications for the precision and stability of the CFA estimates and the identification of latent capability sets. As noted by Tein et al. (2013), class enumeration is influenced by sample size, class separation, and the number of indicators. Although the three-class solution showed satisfactory classification probabilities and theoretically interpretable profiles, uncertainty about the optimal number of classes may remain. Future research using larger samples could establish the stability and replicability of the measurement structure and identified capability sets across different EMS populations.

Furthermore, the dichotomization of the work capability indicators may have influenced the identification and composition of the latent capability sets. Although the threshold was theoretically informed and consistent with previous applications of the instrument, sensitivity analyses using alternative thresholds were not conducted. Consequently, the robustness of the identified capability sets across different operationalizations remains uncertain.

Using a cross-sectional survey design is another limitation as it limits causal inference and prevents examination of changes in work capabilities and flourishing over time. Although the findings were interpreted within the integrated JD-R and CA framework, the proposed processes through which job demands and resources may enable or constrain the realization of work capabilities were not directly examined. Consequently, the observed associations should not be interpreted as evidence of these underlying mechanisms. While cross-sectional studies remain valuable for identifying associations between variables (Andersen et al., 2025), longitudinal research is needed to explore how capability sets develop and change over the course of EMS personnel’s careers. Such designs would also provide a stronger basis for examining the temporal relationships between job demands and resources, the development of work capabilities, and subsequent functioning.

Reliance on self-report measures is another limitation. Although subjective perceptions are central to the SE framework grounded in the CA, self-report data may be susceptible to biases such as social desirability and CMV (Podsakoff et al., 2003; Spector, 2019). Although CMV was considered through supplementary statistical diagnostics, these analyses could not establish the absence or extent of method-related bias. Consequently, CMV remains a potential limitation and may have influenced the observed associations (Johnston et al., 2025). Future studies could strengthen the evidence base by incorporating multiple sources of data, including supervisor ratings, organizational indicators, or objective work-related outcomes where appropriate, and collecting data at different time points.

The quantitative nature of the study further limits insight into the contextual and experiential aspects of capability development. Qualitative research could complement the present findings by exploring how EMS personnel experience and construct their capabilities in practice, particularly in relation to the relatively low levels of enablement and achievement reported for certain work values. Such research may provide a richer understanding of the organizational and contextual factors that facilitate or constrain capability development. It may also help clarify how specific job resources, such as autonomy and co-worker support, are translated into genuine opportunities to achieve valued work outcomes and why particular job demands may constrain capabilities whereas others do not.

Finally, work capabilities were assessed using a predefined set of work values derived from the capability literature. Although these values have demonstrated relevance across occupational groups (Rothmann et al., 2026a), it is possible that additional or context-specific work values are important for EMS personnel in South Africa. Future research could adopt qualitative or mixed-method approaches to identify such values and further refine the application of the capability perspective within emergency service contexts.

## 8 Conclusion

This study examined the relationships between EMS personnel’s job demands, job resources, work capabilities, flourishing at work, and negative affect within the SE model grounded in the CA. By adopting a person-centered perspective, the study identified three distinct work capability sets (weak, constrained, and robust) among EMS personnel, providing a nuanced understanding of how employees differ in their opportunities to achieve valued work outcomes within a demanding and complex healthcare environment in a non-WEIRD context. The identification of these capability sets highlights the importance of considering configurations of valued opportunities rather than individual capabilities in isolation.

The findings highlight the importance of work capabilities for understanding EMS personnel’s flourishing and SE. In particular, autonomy at work and co-worker support emerged as important resources associated with broader capability sets and higher levels of emotional, psychological, and social well-being. Emotional demands, in contrast, were associated with more restricted work capabilities and poorer well-being, while work pressure showed less consistent relationships with capabilities and flourishing but was positively associated with negative affect. These findings suggest that different work conditions may be associated with employees’ opportunities to achieve valued work outcomes and their well-being in different ways.

Overall, the findings suggest that understanding SE requires attention not only to the demands and resources present in the work environment, but also to whether these conditions are associated with genuine opportunities for employees to achieve what they value at work. Integrating the JD-R model with the CA therefore provides a useful framework for understanding how work conditions, substantive opportunities, and valued functioning may be connected. Strengthening enabling work conditions while addressing demands that may restrict employees’ capabilities and well-being may support EMS personnel’s long-term functioning and the sustainability of emergency healthcare services.

## Data Availability

The raw data supporting the conclusions of this article will be made available by the authors, without undue reservation.

## References

[ref1] Abma F. I. Brouwer S. De Vries H. J. Arends I. Robroek S. J. Cuijpers M. P. . (2016). The capability set for work: development and validation of a new questionnaire. Scand. J. Work Environ. Health 42, 34–42. doi: 10.5271/sjweh.3532, 26618990

[ref3] African Union (2026). Linking Agenda 2063 and the SDGs. Available online at: https://au.int/en/agenda2063/sdgs (Accessed August, 15, 2025).

[ref4] Ahmed U. Shah M. H. Siddiqui B. A. Shah S. A. Dahri A. S. Qureshi M. A. (2017). Troubling job demands at work: examining the deleterious impact of workload and emotional demands on work engagement. Int. J. Acad. Res. Bus. Soc. Sci. 7, 96–106. doi: 10.6007/IJARBSS/v7-i6/2949

[ref5] Al-Shaqsi S. (2010). Models of international emergency medical service (EMS) systems. Oman Med. J. 25, 320–323. doi: 10.5001/omj.2010.92, 22043368 PMC3191661

[ref6] Alzahrani A. Keyworth C. Alshahrani K. M. Alkhelaifi R. Johnson J. (2025). Prevalence of anxiety, depression, and post-traumatic stress disorder among paramedic students: a systematic review and meta-analysis. Soc. Psychiatry Psychiatr. Epidemiol. 60, 563–578. doi: 10.1007/s00127-024-02755-6, 39264380 PMC11870987

[ref7] Andersen R. Thomakos D. Wood G. (2025). Common method variance (CMV) bias: its implications, how to detect it, and how to handle it. Hum. Resour. Dev. Int. 1–13. doi: 10.1080/13678868.2025.2537161, 37339054

[ref8] Arjmand H.-A. O’Donnell M. L. Putica A. Sadler N. Peck T. Nursey J. . (2024). Mental health treatment for first responders: an assessment of mental health provider needs. Psychol. Serv. 21, 489–499. doi: 10.1037/ser0000832, 38166296

[ref9] Bakker A. B. (2014). The Job Demands–Resources Questionnaire. Erasmus University.

[ref10] Bakker A. B. Demerouti E. (2024). Job demands–resources theory: frequently asked questions. J. Occup. Health Psychol. 29, 188–200. doi: 10.1037/ocp0000376, 38913705

[ref11] Bakker A. B. Demerouti E. Euwema M. C. (2005). Job resources buffer the impact of job demands on burnout. J. Occup. Health Psychol. 10, 170–180. doi: 10.1037/1076-8998.10.2.170, 15826226

[ref12] Barnard N. B. Rothmann S. De Beer L. T. Lubbe W. (2023a). Burnout of emergency nurses in a south African context: the role of job demands and resources, and capabilities. Front. Psychol. 14:1119063. doi: 10.3389/fpsyg.2023.1119063, 37275737 PMC10233105

[ref13] Barnard N. B. Rothmann S. De Beer L. T. Lubbe W. (2023b). Emergency nurses’ job demands-resources profiles and capabilities: effects on performance and intention to leave. Sustainability 15:5415. doi: 10.3390/su15065415

[ref14] Barnard N. B. Rothmann S. De Beer L. T. Lubbe W. (2023c). Sustainable employability of emergency nurses: the effects of precarious work and mental toughness on capabilities and mental health. J. Nurs. Manag. 2023, 1–17. doi: 10.1155/2023/8840756 PMC11919039 40225661

[ref16] Bolck A. Croon M. Hagenaars J. (2004). Estimating latent structure models with categorical variables: one-step versus three-step estimators. Polit. Anal. 12, 3–27. doi: 10.1093/pan/mph001

[ref17] Bono J. E. Davies S. E. Rasch R. L. (2011). Some Traits Associated with Flourishing at Work. New York, NY: Oxford University Press.

[ref18] Clark L. V. Fida R. Skinner J. Murdoch J. Rees N. Williams J. . (2021). Mental health, well-being and support interventions for UK ambulance services staff: an evidence map, 2000 to 2020. Br. Paramedic J. 5, 25–39. doi: 10.29045/14784726.2021.3.5.4.25, 34421373 PMC8341070

[ref19] Creswell J. W. Creswell J. D. (2020). Research design: Qualitative, Quantitative, and Mixed Methods Approaches. 5th Edn. Thousand Oaks, CA: Sage Publications.

[ref20] Crowe R. P. Fernandez A. R. Pepe P. E. Cash R. E. Rivard M. K. Wronski R. . (2020). The association of job demands and resources with burnout among emergency medical services professionals. J. Am. Coll. Emerg. Physicians Open 1, 6–16. doi: 10.1002/emp2.12014 PMC7493511 33000008

[ref21] Cunningham C. Rosenberg M. Kahle J. (2022). The activities and impacts of a community-based volunteer ambulance service in Cape Town, South Africa. Afr. J. Emerg. Med. 12, 236–241. doi: 10.1016/j.afjem.2022.04.003, 35734546 PMC9194692

[ref9003] De Beer L. T. Schaufeli W. B. Bakker A. B. (2022). Investigating the validity of the short form Burnout Assessment Tool: A job demands-resources approach. African Journal of Psychological Assessment, 4:9. doi: 10.4102/ajopa.v4i0.95

[ref22] De Jonge J. Peeters M. C. W. (2019). The vital worker: towards sustainable performance at work. Int. J. Environ. Res. Public Health 16:910. doi: 10.3390/ijerph16060910, 30871247 PMC6466057

[ref23] De Neve J. E. Ward G. (2025). Why Workplace Wellbeing Matters: The Science behind Employee Happiness and Organizational Performance. Boston, MA: Harvard Business Review Press.

[ref24] De Wet T. Rothmann S. (2022). Toward perceived sustainable employability: capabilities of secondary school teachers in a south African context. Front. Psychol. 13:842045. doi: 10.3389/fpsyg.2022.842045, 35602738 PMC9115101

[ref25] Demerouti E. Bakker A. B. (2011). The job demands–resources model: challenges for future research. S. Afr. J. Ind. Psychol. 37:9. doi: 10.4102/sajip.v37i2.974

[ref26] Demerouti E. Bakker A. B. Nachreiner F. Schaufeli W. B. (2001). The job demands-resources model of burnout. J. Appl. Psychol. 86, 499–512. doi: 10.1037/0021-9010.86.3.499, 11419809

[ref27] Diedericks E. Rothmann S. (2013). Flourishing of information technology professionals: the role of work engagement and job satisfaction. J. Psychol. Afr. 23, 225–233. doi: 10.1080/14330237.2013.10820618

[ref28] Dos Santos Tome J. Van Der Vaart L. (2020). Work pressure, emotional demands and work performance among information technology professionals in South Africa: the role of exhaustion and depersonalisation. S. Afr. J. Hum. Resour. Manag. 18:a1362. doi: 10.4102/sajhrm.v18i0.1362

[ref29] Elmqvist C. Brunt D. Fridlund B. Ekebergh M. (2010). Being first on the scene of an accident – experiences of ‘doing’ prehospital emergency care. Scand. J. Caring Sci. 24, 266–273. doi: 10.1111/j.1471-6712.2009.00716.x, 19732398

[ref30] Elsässer A. Dreher A. Pietrowsky R. Flake F. Loerbroks A. (2024). Psychosocial working conditions, perceived patient safety and their association in emergency medical services workers in Germany – a cross-sectional study. BMC Emerg. Med. 24:62. doi: 10.1186/s12873-024-00983-2, 38616266 PMC11017549

[ref31] Ericsson C. R. Lindström V. Rudman A. Nordquist H. (2022). Paramedics’ perceptions of job demands and resources in Finnish emergency medical services: a qualitative study. BMC Health Serv. Res. 22:1469. doi: 10.1186/s12913-022-08856-9, 36461045 PMC9717484

[ref32] Farcas A. M. Crowe R. P. Kennel J. Little N. Haamid A. Camacho M. A. . (2024). Achieving equity in EMS care and patient outcomes through quality management systems: a position statement. Prehosp. Emerg. Care 28, 871–881. doi: 10.1080/10903127.2024.2352582, 38727731

[ref33] Field A. (2013). Discovering Statistics using IBM SPSS Statistics: and Sex and Drugs and Rock ‘n’ Roll. London, UK: Sage.

[ref35] Gayton S. D. Lovell G. P. (2012). Resilience in ambulance service paramedics and its relationships with well-being and general health. Traumatology 18, 58–64. doi: 10.1177/1534765610396727

[ref36] Geiser C. (2012). Data Analysis with Mplus. New York, NY: The Guilford Press.

[ref37] Gürbüz S. Bakker A. B. Demerouti E. Brouwers E. P. M. (2023). Sustainable employability and work engagement: a three-wave study. Front. Psychol. 14:1188728. doi: 10.3389/fpsyg.2023.1188728, 37397284 PMC10313196

[ref39] Haugen P. T. Evces M. Weiss D. S. (2012). Treating posttraumatic stress disorder in first responders: a systematic review. Clin. Psychol. Rev. 32, 370–380. doi: 10.1016/j.cpr.2012.04.001, 22561967

[ref40] Health Professions Council of South Africa (2026). Emergency care. Available online at: https://www.hpcsa.co.za/board/emergency-care (Accessed March 7, 2026).

[ref41] Henderson A. C. (2013). Examining policy implementation in health care: rule abidance and deviation in emergency medical services. Public Adm. Rev. 73, 799–809. doi: 10.1111/puar.12146

[ref9002] Huang R. Sun H. Chen G. Li Y. Wang J. (2022). Second‐victim experience and support among nurses in mainland China. Journal of Nursing Management, 30:260–267. doi: 10.1111/jonm.13490 34592010

[ref42] Hu L. Bentler P. M. (1999). Cutoff criteria for fit indexes in covariance structure analysis: conventional criteria versus new alternatives. Struct. Equ. Model. 6, 1–55. doi: 10.1080/10705519909540118

[ref43] IBM Corporation (2020). IBM SPSS Statistics for Windows [IBM SPSS Statistics for Windows]. IBM Corporation.

[ref44] Janse Van Rensburg C. Rothmann S. Diedericks E. (2018). Job demands and resources: flourishing and job performance in south African universities of technology settings. J. Psychol. Afr. 28, 291–297. doi: 10.1080/14330237.2018.1501881

[ref45] Johnston S. Waite P. Laing J. Rashid L. Wilkins A. Hooper C. . (2025). Why do emergency medical service employees (not) seek organizational help for mental health support?: a systematic review. Int. J. Environ. Res. Public Health 22:629. doi: 10.3390/ijerph22040629, 40283853 PMC12027444

[ref46] Jones S. Nagel C. McSweeney J. Curran G. (2018). Prevalence and correlates of psychiatric symptoms among first responders in a southern state. Arch. Psychiatr. Nurs. 32, 828–835. doi: 10.1016/j.apnu.2018.06.007, 30454624

[ref47] Karrasch S. Hitzler M. Behnke A. Tumani V. Kolassa I.-T. Rojas R. (2020). Chronic and traumatic stress among emergency medical services personnel: a narrative review on vulnerability factors, resilience, and intervention. Z. Klin. Psychol. Psychother. 49, 204–217. doi: 10.1026/1616-3443/a000600

[ref48] Keyes C. L. M. (2002). The mental health continuum: from languishing to flourishing in life. J. Health Soc. Behav. 43, 207–222. doi: 10.2307/3090197, 12096700

[ref49] Keyes C. L. M. (2007). Promoting and protecting mental health as flourishing: a complementary strategy for improving national mental health. Am. Psychol. 62, 95–108. doi: 10.1037/0003-066X.62.2.95, 17324035

[ref50] Keyes C. L. M. (2024). Languishing: How to Feel Alive Again in a World that Wears us Down. New York, NY: Penguin Random House.

[ref51] Kim J. E. Park J. H. Park S. H. (2019). Anger suppression and rumination sequentially mediates the effect of emotional labor in Korean nurses. Int. J. Environ. Res. Public Health 16:799. doi: 10.3390/ijerph16050799, 30841533 PMC6427706

[ref52] Kirk K. (2024). Time for a rebalance: psychological and emotional well-being in the healthcare workforce as the foundation for patient safety. BMJ Qual. Safety 33, 483–486. doi: 10.1136/bmjqs-2024-017236, 38653549

[ref53] Kline R. B. (2011). Principles and Practice of Structural Equation Modeling. 3rd Edn. New York, NY: The Guilford Press.

[ref54] Krugel L. (2025). South Africa Economic Outlook. Available online at: https://www.strategyand.pwc.com/a1/en/assets/pdf/sa-economic-outlook/navigating-energy-disruptions.pdf (Accessed November 20, 2025).

[ref55] Laguna M. Chilimoniuk B. Purc E. Kulczycka K. (2022). Job-related affective well-being in emergency medical dispatchers: the role of workload, job autonomy, and performance feedback. Adv. Cogn. Psychol. 18, 243–250. doi: 10.5709/acp-0368-x

[ref56] Lesener T. Gusy B. Wolter C. (2019). The job demands-resources model: a meta-analytic review of longitudinal studies. Work Stress. 33, 76–103. doi: 10.1080/02678373.2018.1529065

[ref57] Lo Y. Mendell N. R. Rubin D. B. (2001). Testing the number of components in a normal mixture. Biometrika 88, 767–778. doi: 10.1093/biomet/88.3.767

[ref58] Lochmannová A. Šín R. Kovalčinová K. Marek P. (2026). Workplace stress, psychological distress, and job retention among emergency medical services personnel. J. Work. Behav. Health 41, 949–968. doi: 10.1080/15555240.2025.2565791

[ref60] Maguire B. J. O’Meara P. O’Neill B. J. Brightwell R. (2018). Violence against emergency medical services personnel: a systematic review of the literature. Am. J. Ind. Med. 61, 167–180. doi: 10.1002/ajim.22797, 29178541

[ref61] Malamule D. (2026). MEC for Health Strongly Condemns the criminal Attack on EMS Personnel Available online at: https://www.mpg.gov.za/media/mec-health-strongly-condemns-criminal-attack-ems-personnel (Accessed March, 7, 2026).

[ref62] Marange T. S. Mangwanya M. G. Maramura T. C. Chinyamurindi W. T. (2024). Unlocking mental well-being: the role of workplace social support and organisational justice in the public service. S. Afr. J. Hum. Resour. Manag. 22:a2717. doi: 10.4102/SAJHRM.v22i0.2717

[ref63] Mason P. (2024). Paramedic Flourishing: A way Forward Available online at: https://www.viurrspace.ca/server/api/core/bitstreams/90743451-ac36-4f85-963e-68fec5be594e/content (Accessed August, 15, 2025).

[ref65] Meerman J. Van Casteren P. A. J. Brouwers E. P. M. Van Dam A. Van Der Klink J. J. L. (2022). A capability perspective on sustainable employability: a Dutch focus group study on organizational, work and personal conversion factors. PLoS One 17:e0274000. doi: 10.1371/journal.pone.0274000, 36269699 PMC9586339

[ref9001] Mildenhall J. (2012). Occupational stress, paramedic informal coping strategies: a review of the literature. Journal of Paramedic Practice, 4:318–328. doi: 10.12968/jpar.2012.4.6.318

[ref66] Mishra S. Goebert D. Char E. Dukes P. Ahmed I. (2010). Trauma exposure and symptoms of post-traumatic stress disorder in emergency medical services personnel in Hawaii. Emerg. Med. J. 27, 708–711. doi: 10.1136/emj.2009.080622, 20466825

[ref67] Mnxuma S. L. Rothmann S. Stander M. W. ChaaCha T. (2026). Job demands and resources, work capabilities, and burnout of industrial psychology and human resource management practitioners. Front. Psychol. 17:1755805. doi: 10.3389/fpsyg.2026.1755805 PMC13077283 41987977

[ref68] Murangi A. Rothmann S. Nel M. (2022). Capabilities and work functionings of special education teachers in Namibia. South Afri. J. Indust. Psychol. 48:a2046. doi: 10.4102/sajip.v48i0.2046, 36312118 PMC9606819

[ref69] Murray R. M. Davis A. L. Shepler L. J. Moore-Merrell L. Troup W. J. Allen J. A. . (2020). A systematic review of workplace violence against emergency medical services responders. New Solutions 29, 487–503. doi: 10.1177/1048291119893388, 31841060 PMC8594050

[ref70] Muthén B. O. Muthén L. K. (2017). Mplus User’s Guide: Statistical Analysis with Latent Variables. Los Angeles, CA: Muthén & Muthén.

[ref71] Newton J. Carpenter T. Zwicker J. (2024). Exploring paramedic perspectives on emergency medical service (EMS) delivery in Alberta: a qualitative study. BMC Emerg. Med. 24:66. doi: 10.1186/s12873-024-00986-z, 38627662 PMC11020468

[ref72] Ntatamala I. Adams S. (2022). The correlates of post-traumatic stress disorder in ambulance personnel and barriers faced in accessing care for work-related stress. Int. J. Environ. Res. Public Health 19:2046. doi: 10.3390/ijerph19042046, 35206234 PMC8871647

[ref73] Nunnally J. C. Bernstein I. H. (1994). Psychometric Theory.New York, NY: McGraw-Hill.

[ref74] Petrie K. Milligan-Saville J. Gayed A. Deady M. Phelps A. Dell L. . (2018). Prevalence of PTSD and common mental disorders amongst ambulance personnel: a systematic review and meta-analysis. Soc. Psychiatry Psychiatr. Epidemiol. 53, 897–909. doi: 10.1007/s00127-018-1539-5, 29869691

[ref75] Podsakoff P. M. MacKenzie S. B. Lee J.-Y. Podsakoff N. P. (2003). Common method biases in behavioral research: a critical review of the literature and recommended remedies. J. Appl. Psychol. 88, 879–903. doi: 10.1037/0021-9010.88.5.879, 14516251

[ref77] Ragadu S. C. Rothmann S. (2023). Decent work, capabilities and flourishing at work. Ment. Health Soc. Incl. 27, 317–339. doi: 10.1108/MHSI-05-2023-0054

[ref78] Ragadu S. C. Rothmann S. (2026). Job demands-resources profiles and work capabilities: effects on early childhood development practitioners’ functioning. Early Child. Educ. J. 54, 999–1014. doi: 10.1007/s10643-025-01902-1

[ref79] Rautenbach C. Rothmann S. (2017). Psychometric validation of the flourishing-at-work scale – short form (FWS-SF): results and implications of a south African study. J. Psychol. Afr. 27, 303–309. doi: 10.1080/14330237.2017.1347748

[ref80] Redelinghuys K. Rothmann S. Botha E. (2019). Workplace flourishing: measurement, antecedents and outcomes. S. Afr. J. Ind. Psychol. 45:a1549. doi: 10.4102/sajip.v45i0.1549

[ref81] Regehr C. Millar D. (2007). Situation critical: high demand, low control, and low support in paramedic organizations. Traumatology 13, 49–58. doi: 10.1177/1534765607299912

[ref82] Robeyns I. (2017). Wellbeing, Freedom and Social Justice: The Capability Approach re-Examined. Cambridge, UK: Open Book Publishers.

[ref83] Rojas M. Veenhoven R. (2013). Contentment and affect in the estimation of happiness. Soc. Indic. Res. 110, 415–431. doi: 10.1007/s11205-011-9952-0

[ref84] Rothmann S. (2013). “From happiness to flourishing at work: a southern African perspective,” in Well-Being Research in South Africa, ed. Wissing M. P., vol. 4 (Dordrecht, Netherlands: Springer Netherlands), 123–151. doi: 10.1007/978-94-007-6368-5_7

[ref85] Rothmann S. Barnard N. Nel M. Arendse B. Murangi A. Ragadu S. . (2026a). “Work capabilities in different occupations and countries,” in Capabilities at Work: The Added Value of the Capability Model for Well-Being and Work, eds. Van der Klink J. Rothmann S.. 1st ed (Cambridge, UK: Cambridge University Press), 279–301. doi: 10.1017/9781009600576.017

[ref86] Rothmann S. Buys C. (2011). Job demands and resources, psychological conditions, religious coping and work engagement of reformed church ministers. J. Psychol. Afr. 21, 173–183. doi: 10.1080/14330237.2011.10820446

[ref87] Rothmann S. Ragadu S. Barnard N. Murangi A. (2026b). “The capability model applied to working individuals: decent and flourishing work,” in Capabilities at Work: The Added Value of the Capability Model for Well-Being and Work, eds. Van der Klink J. Rothmann S.. 1st ed (Cambridge, UK: Cambridge University Press), 179–200. doi: 10.1017/9781009600576.012

[ref88] Rothmann S. Van Zyl L. E. Rautenbach C. (2019). “Measuring flourishing @ work interventions: the development and validation of the flourishing-at-work scale,” in Positive Psychological Intervention Design and Protocols for Multi-Cultural Contexts, eds. Van Zyl L. E. Rothmann S. (Cham, Switzerland: Springer International Publishing), 241–276. doi: 10.1007/978-3-030-20020-6_11

[ref90] Safi-Keykaleh M. Khorasani-Zavareh D. Bohm K. (2020). Factors affecting emergency medical technicians’ on-scene decision-making in emergency situations: a qualitative study. Adv. J. Emerg. Med. 4:e88. doi: 10.22114/ajem.v0i0.341

[ref91] Schaufeli W. B. (2017). Applying the job demands-resources model. Organ. Dyn. 46, 120–132. doi: 10.1016/j.orgdyn.2017.04.008

[ref93] Sen A. (1985). Commodities and Capabilities. Amsterdam, Netherlands: Elsevier Science Publications.

[ref94] Sen A. (1999). Development as Freedom. New York, NY: Oxford University Press.

[ref95] Sen A. (2009). The Idea of Justice. Cambridge, UK: Belknap Press of Harvard University Press.

[ref96] Spector P. E. (2019). Do not cross me: optimizing the use of cross-sectional designs. J. Bus. Psychol. 34, 125–137. doi: 10.1007/s10869-018-09613-8

[ref97] Spurk D. Hirschi A. Wang M. Valero D. Kauffeld S. (2020). Latent profile analysis: a review and “how to” guide of its application within vocational behavior research. J. Vocat. Behav. 120:103445. doi: 10.1016/j.jvb.2020.103445

[ref98] Stassen W. Van Nugteren B. Stein C. (2013). Burnout among advanced life support paramedics in Johannesburg, South Africa. Emerg. Med. J. 30, 331–334. doi: 10.1136/emermed-2011-200920, 22505296

[ref99] Struwig F. W. Stead G. B. (2013). Research: Planning, Designing and Reporting. Cape Town, South Africa: Pearson.

[ref100] Tahar Y. B. Rejeb N. Maalaoui A. Kraus S. Westhead P. Jones P. (2023). Emotional demands and entrepreneurial burnout: the role of autonomy and job satisfaction. Small Bus. Econ. 61, 701–716. doi: 10.1007/s11187-022-00702-w, 38625348 PMC9589686

[ref101] Tein J.-Y. Coxe S. Heining C. (2013). Statistical power to detect the correct number of classes in latent profile analysis. Struct. Equ. Model. Multidiscip. J. 20, 640–657. doi: 10.1080/10705511.2013.824781, 24489457 PMC3904803

[ref102] The jamovi project (2024). The jamovi project [The jamovi project]. Available online at: https://www.jamovi.org

[ref103] Thielmann B. Schnell J. Böckelmann I. Schumann H. (2022). Analysis of work related factors, behavior, well-being outcome, and job satisfaction of workers of emergency medical service: a systematic review. Int. J. Environ. Res. Public Health 19:6660. doi: 10.3390/ijerph19116660, 35682241 PMC9180048

[ref104] Thielmann B. Schwarze R. Böckelmann I. (2023). A systematic review of associations and predictors for job satisfaction and work engagement in prehospital emergency medical services—challenges for the future. Int. J. Environ. Res. Public Health 20:4578. doi: 10.3390/ijerph20054578, 36901586 PMC10002026

[ref105] Tiwari R. Naidoo R. English R. Chikte U. (2021). Estimating the emergency care workforce in South Africa. Afr. J. Primary Health Care Family Med. 13, e1–e9. doi: 10.4102/phcfm.v13i1.3174, 34879696 PMC8678951

[ref106] Tummers L. G. Bakker A. B. (2021). Leadership and job demands-resources theory: a systematic review. Front. Psychol. 12:722080. doi: 10.3389/fpsyg.2021.722080, 34659034 PMC8514935

[ref108] United Nations (2015). The 17 Sustainable Development Goals, Available online at: https://sdgs.un.org/goals (Accessed August, 15, 2025).

[ref109] Van der Klink J. J. Bültmann U. Burdorf A. Schaufeli W. B. Zijlstra F. R. Abma F. I. . (2016). Sustainable employability – definition, conceptualization, and implications: a perspective based on the capability approach. Scand. J. Work Environ. Health 42, 71–79. doi: 10.5271/sjweh.3531, 26595874

[ref110] Van der Klink J. J. Rothmann S. (2026). “The capability model for work: an introduction,” in Capabilities at Work: The Added Value of the Capability Model for Well-Being and Work, eds. Van der Klink J. Rothmann S.. 1st ed (Cambridge, UK: Cambridge University Press), 3–17. doi: 10.1017/9781009600576.003

[ref111] Van der Klink J. Schaufeli W. Bültmann U. Brouwer S. Abma F. Burdorf A. . (2026). “Capabilities and work: new developments towards sustainability and well-being at work,” in Capabilities at Work: The Added Value of the Capability Model for Well-Being and Work, eds. Van der Klink J. Rothmann S.. 1st ed (Cambridge, UK: Cambridge University Press), 18–52. doi: 10.1017/9781009600576.004

[ref112] Van der Vaart L. Blonk R. Boermans S. Schaufeli W. B. Welch Saleeby P. (2026). “Integrating the capability approach with other theories of work and employee health and well-being,” in Capabilities at Work: The Added Value of the Capability Model for Well-Being and Work, eds. van der Klink J. Rothmann S.. 1st ed (Cambridge, UK: Cambridge University Press), 116–140. doi: 10.1017/9781009600576.008

[ref113] Van Puyvelde M. Van Herck J. Van Den Bossche J. Goethals F. Gijbels D. Detaille F. . (2023). Walk the line: a systemic perspective on stress experienced by emergency medical personnel by comparing military and civilian prehospital settings. Front. Public Health 11:1136090. doi: 10.3389/fpubh.2023.1136090, 37441639 PMC10335750

[ref114] Wallace J. B. (2026). Mattering: The Secret to Building a Life of Deep Connection and Purpose. New York, NY: Harper Collins Publishers.

[ref116] Wang J. Wang X. (2020). Structural Equation Modeling: Applications using Mplus. Hoboken, NJ: Wiley.

[ref117] Wende H. (2024) “It Isn’t safe”: South African first Responders Attacked while Saving lives. Al Jazeera. Available online at: https://www.aljazeera.com/features/2024/12/4/it-isnt-safe-south-african-first-responders-attacked-while-saving-lives (Accessed March, 7, 2026).

[ref118] West S. G. Taylor A. B. Wu W. (2012). “Model fit and model selection in structural equation modeling,” in Handbook of Structural Equation Modeling, ed. Hoyle R. H. (New York, NY: The Guilford Press), 209–231.

[ref119] Wijk C. V. Cronje F. Meintjes J. (2020). Mental wellbeing monitoring in a sample of emergency medical service personnel. Occup. Dis. Environ. Med. 8, 26–33. doi: 10.4236/odem.2020.81002

[ref120] Wojtysiak K. Wielgus A. Zielińska-Więczkowska H. (2021). The impact of working conditions on stress and its effects in the group of paramedics: a systematic review. Humanities & Social Sciences Reviews 9, 188–193. doi: 10.18510/hssr.2021.9426

